# Magnetic Mesoporous Silica Nanorods Loaded with Ceria and Functionalized with Fluorophores for Multimodal Imaging

**DOI:** 10.1021/acsanm.1c03837

**Published:** 2022-02-10

**Authors:** Jan Grzelak, Jaume Gázquez, Alba Grayston, Mariana Teles, Fernando Herranz, Nerea Roher, Anna Rosell, Anna Roig, Martí Gich

**Affiliations:** † Institut de Ciència de Materials de Barcelona (ICMAB-CSIC), Campus UAB, 08193 Bellaterra, Catalonia, Spain; ‡ Neurovascular Research Laboratory, Vall d’Hebron Research Institute (VHIR), 08035, Barcelona, Catalonia, Spain; § Institute of Biotechnology and Biomedicine (IBB), Universitat Autònoma de Barcelona, 08193 Bellaterra, Catalonia, Spain; ∥ Instituto de Química Médica (IQM), Consejo Superior de Investigaciones Científicas (CSIC), 28006 Madrid, Spain

**Keywords:** mesoporous silica rods, superparamagnetic nanoparticles, magnetic resonance imaging, fluorescence imaging, multimodal nanoparticles, anisotropic nanoparticles

## Abstract

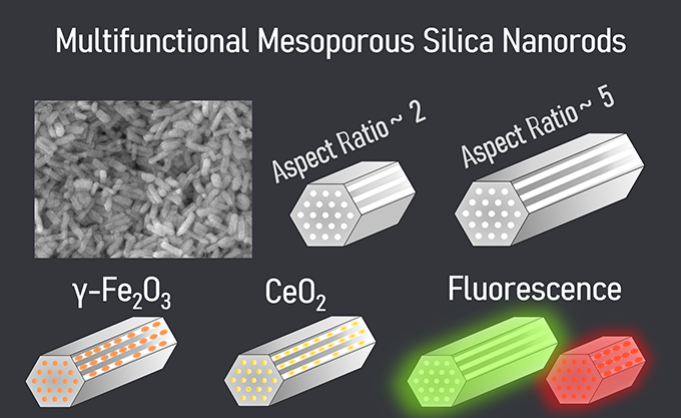

Multifunctional magnetic nanocomposites based on mesoporous silica have a wide range of potential applications in catalysis, biomedicine, or sensing. Such particles combine responsiveness to external magnetic fields with other functionalities endowed by the agents loaded inside the pores or conjugated to the particle surface. Different applications might benefit from specific particle morphologies. In the case of biomedical applications, mesoporous silica nanospheres have been extensively studied while nanorods, with a more challenging preparation, have attracted much less attention despite the positive impact on the therapeutic performance shown by seminal studies. Here, we report on a sol–gel synthesis of mesoporous rodlike silica particles of two distinct lengths (1.4 and 0.9 μm) and aspect ratios (4.7 and 2.2) using Pluronic P123 as a structure-directing template and rendering ∼1 g of rods per batch. Iron oxide nanoparticles have been synthesized within the pores yielding maghemite (γ-Fe_2_O_3_) nanocrystals of elongated shape (∼7 nm × 5 nm) with a [110] preferential orientation along the rod axis and a superparamagnetic character. The performance of the rods as T_2_-weighted MRI contrast agents has also been confirmed. In a subsequent step, the mesoporous silica rods were loaded with a cerium compound and their surface was functionalized with fluorophores (fluorescamine and Cyanine5) emitting at λ = 525 and 730 nm, respectively, thus highlighting the possibility of multiple imaging modalities. The biocompatibility of the rods was evaluated in vitro in a zebrafish (*Danio rerio*) liver cell line (ZFL), with results showing that neither long nor short rods with magnetic particles caused cytotoxicity in ZFL cells for concentrations up to 50 μg/ml. We advocate that such nanocomposites can find applications in medical imaging and therapy, where the influence of shape on performance can be also assessed.

## Introduction

Nanocomposites with unique properties and synergistic actions from different components have a great potential to be used in fields such as biomedicine,^1,2^ catalysis,^3,4^ and energy.^5,6^ Mesoporous silica nanocomposites are mechanically and thermally stable, present large surface areas and pore volumes with narrow pore size distributions, and their particle and pore sizes can be controlled.^7−14^ These characteristics endorse mesoporous silica as an attractive platform to design nanocomposites by loading the pores with different chemical species. Such materials can encapsulate large amounts of nanoscopic cargo while maintaining their robustness and pore ordering. Among a variety of silica mesophases, Santa Barbara Amorphous-15 (SBA-15) is one of the most extensively studied because of its highly ordered and hexagonally arranged cylindrical pores with tunable size between ∼4 and 30 nm.^7,15−17^

The performance of mesoporous silica nanocomposites can be adjusted by controlling the size and porosity of silica, the amount and chemical character of the cargo(s), and the surface chemistry of the composites. For instance, nanocomposites with magnetic particles benefit from a response to external magnetic fields, and can be combined with additional functionalities by hosting other chemical species. This can be very useful for applications such as site-specific cargo delivery,^18,19^ magnetic resonance imaging (MRI),^20−22^ magnetically switchable catalytic reactions,^23−25^ sensing combined with magnetic separation,^26−29^ recycling of magnetic carriers,^30,31^ or the development of catalytic nanomotors.^32^ In particular, mesoporous silica particles incorporating different moieties can be of interest in multimodal contrast agents for medical imaging, combining the advantages of each imaging technique and improving the diagnostics of diseases.^33^ A significant number of studies have been devoted to the biomedical applications of spherical mesoporous silica particles,^34−38^ but much less research considered other specific shapes (only ∼10% concern rods). Indeed, preparing silica in well-defined asymmetric morphologies is extremely challenging, as it requires stringent control of the synthesis conditions. But it appears that these experimental efforts would pay off. Indeed, pioneering studies focusing on rodlike particles have shown increased performance in relevant aspects, such as cell uptake, toxicity, degradation, or drug release,^39−47^ and it has been suggested that the efficient uptake of anisotropic nanoparticles could mimic the advantage of rod-shaped bacteria for internalization in nonphagocytic cells.^48^

Following these ideas, in the present study, we report on the synthesis of mesoporous silica rods intended for multimodal imaging and therapeutic action using biocompatible materials from the design phase. We have developed two different highly reproducible synthetic protocols to prepare rods of two distinct aspect ratios with narrow size distributions. The silica pores were loaded with magnetic γ-iron oxide (maghemite) particles following a solvent-free impregnation method.^9^ Silica powders were mixed with molten iron nitrate, which could flow into the pores by capillarity. The impregnation was followed by a thermal treatment to form iron oxide nanocrystals of around 7 nm in length inside the pores. The possibility of material multimodality is demonstrated by additionally impregnating the rods with cerium (IV) oxide, a material with excellent antioxidant capacity recently reported as a potential therapeutic agent in liver inflammation,^49,50^ and modifying the silica surface with two fluorophores emitting in two distinct spectral ranges (525 and ∼730 nm). Additionally, the biocompatibility of the rods was evaluated using the ZFL cell line as an in vitro model. We believe that such versatile materials could potentially be used in different fields in the near future, especially as multimodal agents in biomedicine.

## Materials and Methods

### Materials

Mesoporous silica rods (MSR) were synthesized using the following reagents. Hydrochloric acid (37%), poly(ethylene glycol)-block-poly(propylene glycol)-block-poly(ethylene glycol) (Pluronic P123), tetraethyl orthosilicate (TEOS), iron (III) nitrate nonahydrate, (3-aminopropyl)triethoxysilane (APTES), agarose, sodium hydroxide, sodium citrate, fluorescamine, and triethylamine were purchased from Sigma-Aldrich and used as received. Ethanol was purchased from PanReac. Iron (III) nitrate nonahydrate and cerium (III) nitrate hexahydrate were purchased from Acros Organics and used as received. Cyanine5 NHS ester (Cy5-NHS) was purchased from Lumiprobe and used as received. The purity of all reagents was 98% or higher.

Zebrafish liver (ZFL) cells were cultured at 28 °C in 5% CO_2_ in Dulbecco’s modified Eagle’s medium (DMEM) with 4.5 g/L glucose, supplemented with 0.01 mg/mL insulin, 50 ng/mL EGF, 5% (v/v) antibiotic/antimycotic solution, 10% (v/v) heat inactivated fetal bovine serum (FBS), and 0.5% (v/v) heat inactivated trout serum (TS) as described in the literature.^51^ The MTT (3-(4,5-dimethylthiazol-2-yl)-2,5-diphenyltetrazolium bromide) substrate and dimethyl sulfoxide (DMSO) were purchased from Sigma-Aldrich.

### Synthesis of Silica Rods with a High Aspect Ratio (Long Rods, LR)

In the first step of the study, mesoporous silica rods of two aspect ratios were synthesized. In a typical synthesis, adapted from the literature,^9,10,52^ 2 g of Pluronic P123 was dissolved in 95 mL of 1.7 M hydrochloric acid solution contained in a reaction bottle of 250 mL capacity (Simax). After obtaining a homogeneous solution, the temperature was increased to 40 °C and the solution was stirred at 700 rpm with a magnetic stirring bar (dimensions: 25 mm × 6 mm). After 3 h, 4.2 g of tetraethyl orthosilicate (TEOS) were added dropwise. Stirring was stopped after 5 min, the reaction bottle was hermetically closed with a plastic cap, and the reaction was kept under static conditions for 24 h. After that, the reaction bottle was transferred to an oven at 80 °C and kept there for 24 h. In a subsequent step, silica was filtered and dried at 55 °C overnight. The surfactant was removed by washing the product in ethanol (24–40 cycles of a 500 mL soxhlet). Then, the material was calcined in air at 550 °C for 5 h. One batch synthesis typically yielded approximately 1 g.

### Synthesis of Silica Rods with a Low Aspect Ratio (Short Rods, SR)

Pluronic P123 (2.4 g) was dissolved in 80 mL of 2 M hydrochloric acid solution in a 250 mL bottle (Simax). After obtaining a homogeneous solution, the temperature was increased to 40 °C and the solution was stirred at 700 rpm using a magnetic stirring bar (dimensions: 25 mm × 6 mm). After 3 h, 5.1 g of TEOS was added dropwise. Stirring was stopped after 4 min and the reaction was kept under static conditions for 3 h. Then, the reaction bottle was transferred to an oven at 80 °C and kept there for 24 h. Then, silica was filtered and dried at 55 °C overnight. The surfactant was removed by washing the product in ethanol (24–40 cycles of a 500 mL soxhlet). Finally, the material was calcined in air at 550 °C for 5 h. One batch synthesis typically yielded approximately 1.2 g.

### Synthesis of Iron Oxide Nanoparticles Inside Silica Pores

The impregnation of mesoporous silica rods consisted of mixing the silica powder with iron (III) nitrate nonahydrate. To induce capillary action, it was made sure that the volume of the iron precursor *V*_Fe(NO_3_)_3_·9H_2_O_ = (*m*_Fe(NO_3_)_3_·9H_2_O_)/(ρ_Fe(NO_3_)_3_·9H_2_O_) was not larger than the volume of the pores (calculated from BET isotherm; 0.65 cm^3^/g for LR, 0.79 cm^3^/g for SR). In a typical impregnation experiment, the powders were mixed at a ratio of 1:1 wt/wt. Iron (III) nitrate nonahydrate was ground in a mortar, weighed, and added to a vial containing the required amount of MSRs. The two powders were mixed thoroughly with a spatula and the vial was put in a silicone oil bath at 60 °C. After 30 min, the powders were mixed again. This step was performed twice. After another 30 min of heating, the powder was cooled down. Then, the impregnated silica was heated in a tubular furnace (ST 1002540, HOBERSAL) at 425 °C for 3 h under a flow of argon with 5% of hydrogen (v/v; 100 cm^3^/min). Rods were labeled as Fe_2_O_3_@LR and Fe_2_O_3_@SR.

### Synthesis of Ceria and Iron Oxide Nanoparticles Inside Silica Pores

The MSR powder was mixed with cerium (III) nitrate hexahydrate (3:1 wt/wt). The cerium precursor was ground in a mortar, weighed, and added to a vial containing MSRs. The two powders were mixed thoroughly with a spatula, and the vial was put in a silicone oil bath at 80 °C. After 30 min, the powders were mixed again. This step was performed twice. After another 30 min of heating, the powder was cooled down. Then, iron (III) nitrate nonahydrate ground in a mortar was added (*m*_iron precursor_ = *m*_cerium precursor_). The powders were mixed thoroughly, and the vial was put in a silicone oil bath at 60 °C. After 30 min, the powders were mixed again. This step was performed twice. After another 30 min of heating, the powders were cooled down. The impregnated MSR were heated in a furnace (ST 1002540, HOBERSAL) at 600 °C for 3 h under a flow of argon with 5% of hydrogen (v/v; 100 cm^3^/min).

### Surface Functionalization with Amine Groups

Amine groups were introduced to the silica rod surface by aminosilanization with APTES, in a protocol that was adapted from the literature^53,54^ with modifications. A solution of ethanol in water (70:30 v/v) was prepared and heated to 70 °C. MSRs (20 mg) were dispersed in 20 mL of this solution by ultrasonication. APTES (120 μL) was added to the dispersion, and the mixture was kept at 70 °C under reflux for 1 h. The particles were then washed three times in ethanol and centrifuged at 16 773*g*. Then, the particles were dispersed in a small amount of ethanol and dried at 60 °C. This step was followed by heating at 120 °C for 2 h in air.

### MSR Functionalization with Fluorescamine and Cyanine5

In a typical synthesis, a stock solution was prepared by dissolving 1.4 mg of fluorescamine in 4 mL of acetone. Amino-functionalized MSRs (5 mg) were dispersed in 2.5 mL of acetone and sonicated in an ultrasound bath. Then, 2.5 mL of the fluorescamine stock solution was added to the dispersion. The mixture was left to react overnight at room temperature, in the dark, under magnetic stirring. Then, the particles were purified by washing three times with ethanol and centrifugation (16 773*g*, 8 min). Finally, the precipitate was dispersed in a small amount of acetone and dried in vacuum. The product was stored at 4 °C in the dark.

A Cyanine5 solution was prepared by dissolving 1 mg of Cyanine5 NHS ester in 5 mL of acetone. Amino-functionalized MSRs (10 mg) were dispersed in the solution by sonication in an ultrasound bath. Then, 80 μL of trimethylamine was added. The mixture was left to react overnight at room temperature, in the dark, under magnetic stirring. Then, the particles were purified by washing three times with ethanol and centrifugation (16 773*g*, 8 min). Finally, the precipitate was dispersed in a small amount of acetone and dried in vacuum. The product was stored at 4 °C in the dark.

### Characterization of Silica Rods

The morphology of MSRs was examined in a JEOL 1210 transmission electron microscope (TEM) operating at 120 kV accelerating voltage. Samples for TEM analysis were prepared by drop-casting ethanol dispersions of the particles (1 mg/mL) on a holey carbon film supported on a copper grid.

The size distributions were obtained using ImageJ software^55^ from a statistical number of the particles (*n* = 300) from several frames acquired from different regions of the sample.

A field emitting scanning electron microscope (SEM, FEI Quanta 200 FEG, Thermo Fisher Scientific, Oregon) was used to study the morphologies of silica particles. The images were acquired in high vacuum and with an accelerating voltage of 10 kV. The samples were prepared by covering a carbon tape attached to a metal SEM holder with a small amount of silica powder. The unattached excess of powder was then removed.

Nitrogen adsorption–desorption isotherms were measured at 77 K using Micromeritics ASAP 2000 apparatus in standard operating mode. Before analysis, all samples were degassed for 20 h at 180 °C under vacuum (*p* < 2 × 10^–3^ Torr). Pore size distribution was calculated from the Barrett–Joyner–Halenda (BJH) model from the desorption branch. The Brunauer–Emmett–Teller (BET) specific surface area was obtained in the 0.05–0.30*p*/*p*_0_ range.

Small-angle X-ray scattering (SAXS) measurements were performed using Bruker D8-Discover in 2θ range from 0.15 to 5° in steps of 0.02° with Cu Kα radiation (λ = 1.5406 Å). A lead plate was placed perpendicular to the sample plane, slightly above it, and held with a specific support designed to minimize the background at the detector.

Thermogravimetric analysis (TGA) was performed by measuring around 2 mg of silica rods sample in a SETSYS Evolution TGA (Setaram) from room temperature to 650 °C at a heating rate of 10 °C/min and under dynamic dry airflow.

The colloidal stability of the rods can only be qualitatively evaluated by visual inspection because measuring the hydrodynamic size by dynamic light scattering is not possible for these systems since the software used to determine the size assumes spherical shapes. The rods remain colloidally dispersed in an aqueous solution for about 30 min before starting to precipitate. They can be easily redispersed by a gentle sonication and the addition of a small amount of mannitol slows down the sedimentation.

A Siemens D-5000 diffractometer in Bragg–Brentano geometry with conventional X-ray source (Cu Kα radiation, λ = 1.5406 Å) was used for X-ray diffraction measurements, which were analyzed by Rietveld refinement using the Materials Using Diffraction (MAUD) software.^56^

The scanning transmission electron microscopy/electron energy loss spectroscopy (STEM/EELS) measurements were performed at the *ICTS National Center of Electron Microscopy* at *Universidad Complutense de Madrid*. Samples were characterized using a JEOL JEM ARM200cF operated at 200 kV equipped with a CEOS aberration corrector and with a Gatan Quantum energy filter spectrometer for EELS measurements. The STEM microscope was operated in high angle annular dark-field (HAADF) imaging mode, also referred to as Z-contrast because the brightness associated with each atomic column of the images scales with its atomic number.^57^ The samples were prepared, as described above, by dispersing the samples in ethanol and depositing a droplet of the suspension on a holey carbon film supported on a copper grid.

Magnetic measurements were performed in a superconducting quantum interference magnetometer (SQUID, Quantum Design, Inc.) The magnetization at room temperature was measured up to a maximum applied field of 50 kOe. The temperature dependence of the magnetization (M-T) under a dc field of 50 Oe was recorded on heating at 2 K/min after zero-field-cooling (ZFC) and field-cooling (FC) conditions in the 10–300 K temperature range.

The iron content in the samples was determined by inductively coupled plasma - optical emission spectrometry (ICP-OES) at the chemical analysis service at *Universitat Autònoma de Barcelona*. Approximately 1 mg of sample (weighed on an MX5 microbalance, Mettler Toledo) was digested with a mixture of concentrated HCl and HNO_3_ (2:1 v/v) in a microwave oven. The resulting digestions were diluted with 1% HCl (v/v) and introduced into an ICP-OES spectrometer (PerkinElmer, model Optima 4300 DV) for the measurement.

The MRI studies on phantoms were performed at the *Institute of Medical Chemistry* (IQM-CSIC, Madrid). The MRI equipment used in this study was an Agilent/Varian scanner (Agilent, Santa Clara, CA) equipped with a DD2 console and an actively shielded 205/120 gradient insert coil with 130 mT m^–1^ maximum gradient strength, a TX/RX volume quadrature coil, and a 1H circularly polarized transmit–receive volume coil of 35 mm inner diameter and 30 mm active length built by Neos Biotec (Pamplona, Spain). The scans were performed at 7 T.

ζ potential values of silica rods before and after surface functionalization were measured by Zetasizer Nano ZS (Malvern). MSRs were dispersed in distilled water at a concentration of 0.5 mg/mL and placed in a ζ potential cuvette for the measurements.

Fourier transform infrared spectra were measured by an FT-IR spectrometer (FT-IR JASCO 4700LE) in transmittance mode in a range from 4000 to 400 cm^–1^, using a circular pellet made of sample powder mixed with KBr.

The XPS analysis was performed at the *Advanced Microscopy Laboratory* (LMA) unit at the *Universidad de Zaragoza*. The measurements were performed with Kratos AXIS Supra XPS spectrometer using a monochromatic X-ray source (Al Kα 120 W, 8 mA/15 kV) under a pressure of 10^–9^ Torr. The size of the area analyzed was 2 mm × 1 mm. The samples in powder form were placed together on a holder with a conducting double-sided tape to be analyzed under the same conditions. Because the samples under investigation were magnetic, the magnetic lenses of the spectrometer could not be used and the measurements were performed in electrostatic mode. It was necessary to employ a charge neutralizer and optimize its parameters to the samples because a strong effect of differential charge was observed could not be neutralized with the default parameters of the equipment. For each sample, a general scan was performed, followed by high-resolution acquisitions in the following regions of interest: C 1s, N 1s, O 1s, and Si 2p.

The fluorescence spectra were recorded with an LS 45 spectrofluorometer (PerkinElmer). The samples were dispersed in acetone at a concentration of 1 mg/mL and placed in a quartz cuvette. The emission spectra of fluorescamine-functionalized samples were recorded for an excitation wavelength λ = 390 nm.

The UV–vis spectra were collected on an Infinite M200PRO Microplate Reader (TECAN) working in absorbance mode. Dispersions of MSRs in acetone at 1 mg/mL were prepared in 96-well plates (Thermo Fisher).

Fluorescence molecular imaging (FMI) studies were performed at the Pre-clinical imaging Platform of *Vall d’Hebron Institut de Recerca* (VHIR, Barcelona). The fluorescence of MSRs was characterized using an IVIS Lumina LT Series III imaging system (PerkinElmer, Waltham, MA). All images were acquired at the following λ_ex_/λ_em_ ranges: 625–655/695–770 nm, centered at 640/732 nm, respectively. A series of concentrations of Cyanine5-functionalized short silica rods with and without Fe_2_O_3_ nanoparticles (respectively, labeled Fe_2_O_3_@SR-Cy5 and SR-Cy5) dispersed in a D-mannitol aqueous solution (3 mg/mL) were prepared in saline (1.5, 1, 0.75, 0.5, 0.25, and 0.1 mg/mL), and duplicates of 100 μL per well were imaged in a 96-well plate. Background wells containing saline (0 mg/mL) were used for background measurements. The fluorescence was measured as total radiant efficiency—TRE ([photons/s]/[μW/cm^2^]). For quantification, circular regions of interest (ROIs) were manually drawn surrounding the fluorescence signal and TRE was measured using the Living Image software (PerkinElmer, Waltham, MA) and corrected by the TRE from the corresponding ROI in the background control well.

### ZFL Cell Viability Studies

Cytotoxic effects of MSRs on ZFL were assessed using the MTT assay. The studies were performed at the *Institute of Biotechnology and Biomedicine (IBB, UAB).* After 2 h in minimal medium (0–0.5% FBS; 2% antibiotic/antimycotic), cultures were incubated with MSRs (Fe_2_O_3_@LR, Fe_2_O_3_@SR, each MSR type and condition in triplicate). 10% of culture medium volume was removed and replaced with the same volume of either water or stock solution of MSRs, to obtain MSR concentrations of 0, 5, 20, 50, and 200 μg/mL. The cells were incubated with MSRs for 6 h at 28 °C. The cells were then washed with PBS, and the MTT substrate was added to 10% of the total volume and further incubated at 28 °C for 30 min. The solution was removed, the cells were solubilized in DMSO, and the lysate absorbance was read on a Victor 3 plate reader (PerkinElmer) at 550 nm. The experiment was repeated four times for each type of MSRs. Data were normalized using GraphPad Prism (version 7.01 for Windows, GraphPad Software, San Diego, California USA, www.graphpad.com) such that the control readings were set at 100%. Two-way ANOVA was performed with Dunnett’s multiple comparison test, comparing treatment and control means.

### Safety

Silica powders used in this work were airborne. Data available for different silica materials indicate that such materials can accumulate in the lungs when inhaled, leading to adverse effects on health, such as silicosis.^58^ For this reason, special protective measures were taken while working with silica in powder form. Sample manipulation was performed exclusively in a fume hood. FFP3 masks (3M Aura 9300/Handanhy HY9330/Labbox) were used by every person present in the laboratory at the time of manipulation, and double nitrile gloves were used for working with silica. The interior of the fume hood was then carefully cleaned with ethanol and all material contaminated with silica was disposed of in an appropriate solid residue container.

## Results and Discussion

Mesoporous silica rods (MSRs) of SBA-15 type were synthesized using a sol–gel method (Figure S1) with Pluronic P123 surfactant as a structure-directing template.^7,52^ This template was later removed by washing with ethanol and calcination.

**Figure 1:**
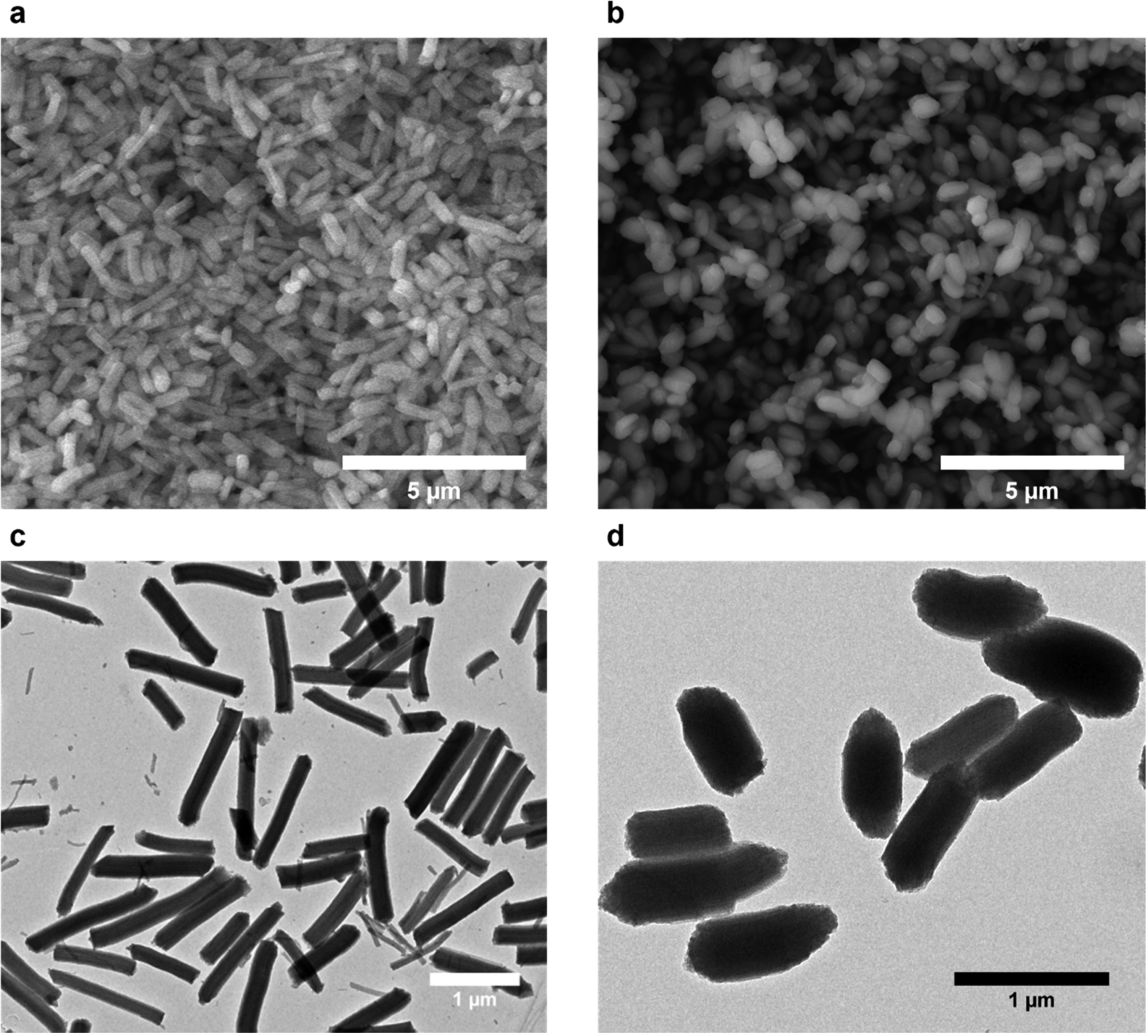
SEM images of (a) long and (b) short rods. TEM images of (c) long and (d) short rods.

As it has been previously reported in the literature,^52^ the silica morphology depends greatly on the synthesis conditions, and we have observed different particle shapes for different temperatures and stirring rates (Figure S2 and Table S1). Once establishing the optimal temperature and stirring rate for obtaining rodlike particles, these parameters were kept constant, and other parameters, namely, the acid concentration, the surfactant concentration, and the reaction mixture volume, were varied. The SEM and TEM images of Figure 1 show the morphologies of the two types of silica rods with distinct aspect ratios (AR) resulting from the optimization of two synthetic protocols. These are denominated as long rods (LR, AR = 4.7, Figure 1a,c) and bacteria-like short rods (SR, AR = 2.2, Figure 1b,d). The SEM images show a large number of particles with a high degree of size uniformity (Figure 1a,b). In the TEM images, the rodlike shape of the particles is clearly evidenced (Figure 1c,d). TEM images were used to characterize the length and width of MSRs, and the measured mean values are shown in Table 1.

**Table 1:** Morphologies of Silica Rods

sample ID	length (μm)	width (μm)	aspect ratio
LR	1.4 ± 0.3	0.3 ± 0.1	4.7 ± 1.3
SR	0.9 ± 0.1	0.4 ± 0.1	2.2 ± 0.7

Studying the results using a factorial design,^59^ we found that within the studied range of variable values, the acid concentration was the critical parameter determining the AR of the rods, as a higher HCl concentration promoted the formation of shorter rods (see Table S2).

**Figure 2:**
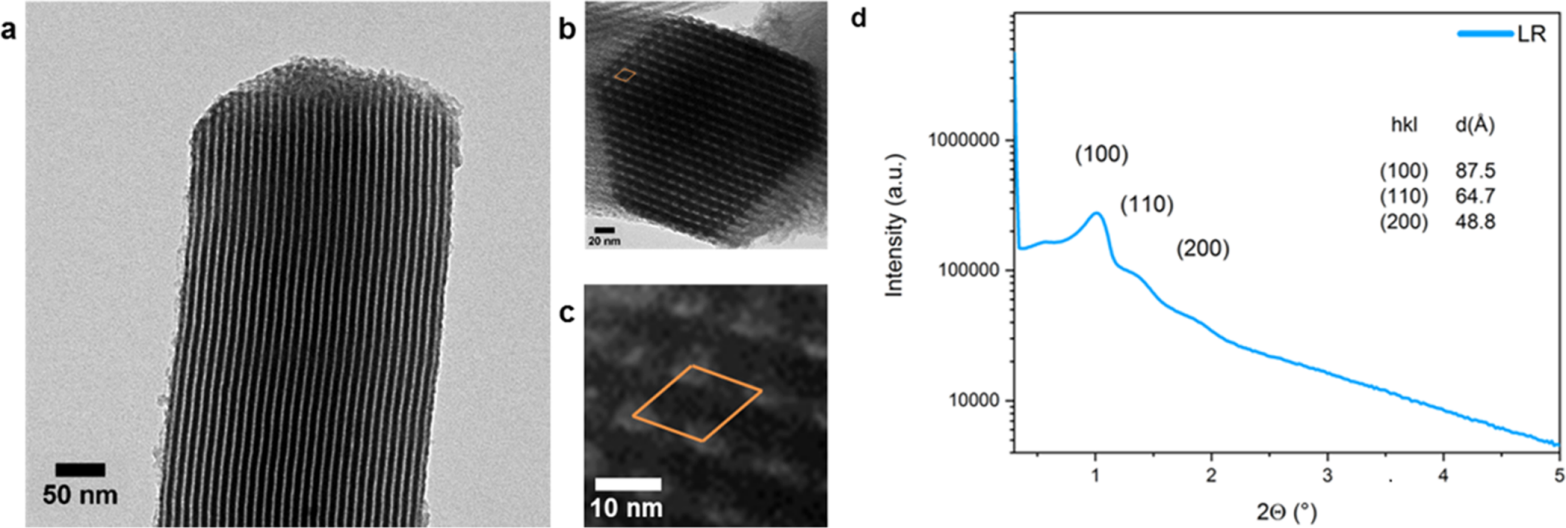
(a) TEM image showing the pores inside a silica rod. Silica walls are seen darker and the parallel channels as bright lines. (b) Cross section of a silica rod showing a hexagonal arrangement of the pores. A unit cell is marked in orange. (c) Magnification of the image showing the unit cell. (d) SAXS pattern of LR. Scattering reflections are indexed in a hexagonal unit cell of the *p*6*mm* space group.

The rods present an array of hexagonally ordered, well-defined mesopores of approximately 5 nm, as observed by TEM (Figure 2a–c). The pores can be seen as bright domains, separated by darker walls. The size of the pores spacing observed in TEM (approximately 10 nm) was compared with the lattice parameter obtained from small-angle X-ray scattering (SAXS, Figure 2d). The scattering reflections at low angles are the ones expected from hexagonal pore ordering (*p*6*mm* space group) previously reported for SBA-15.^7^ The calculated lattice parameter (*a* = 10.1 nm) was in agreement with the sizes measured by TEM.

The Pluronic P123 surfactant removal upon ethanol cleaning and calcination was confirmed by thermogravimetry analysis (Figure S3) and the increased porosity was further confirmed by nitrogen adsorption–desorption isotherms (Figure S4a–c). Both types of rods were characteristic of type-IV isotherms with H1 type hysteresis,^60^ corresponding to mesopores with a narrow pore size distribution. The total surface area, pore volume, and pore diameter values calculated from the isotherms are presented in Table S3. LR and SR had surface areas of up to 827 and 672 m^2^/g, respectively, and a total specific pore volume of up to 1 cm^3^/g. Pore sizes obtained from BET isotherm were in good agreement with values observed in TEM (approximately 5 nm). The calcination slightly decreased the surface area and pore volume as a result of the densification which silica undergoes at high temperatures,^61^ but the mesopore structure remained intact, as seen by TEM (Figure S5).

In a subsequent step, magnetic nanoparticles were synthesized inside the pores by a wet impregnation method. The molten salt (iron (III) nitrate nonahydrate) was incorporated inside the pores by capillary forces. The conversion of the salt to iron oxide was performed by thermal treatment of 3 h at 425 °C in an Ar/H_2_ atmosphere. The samples obtained are referred to as Fe_2_O_3_@LR and Fe_2_O_3_@SR, respectively, and were used in further experiments. The first crystallization attempts done at the same temperature in an air atmosphere resulted in the undesired formation of a mixture of maghemite and the weakly magnetic hematite, which is detrimental to its use as an MRI contrast agent (see Figure S6).

**Figure 3:**
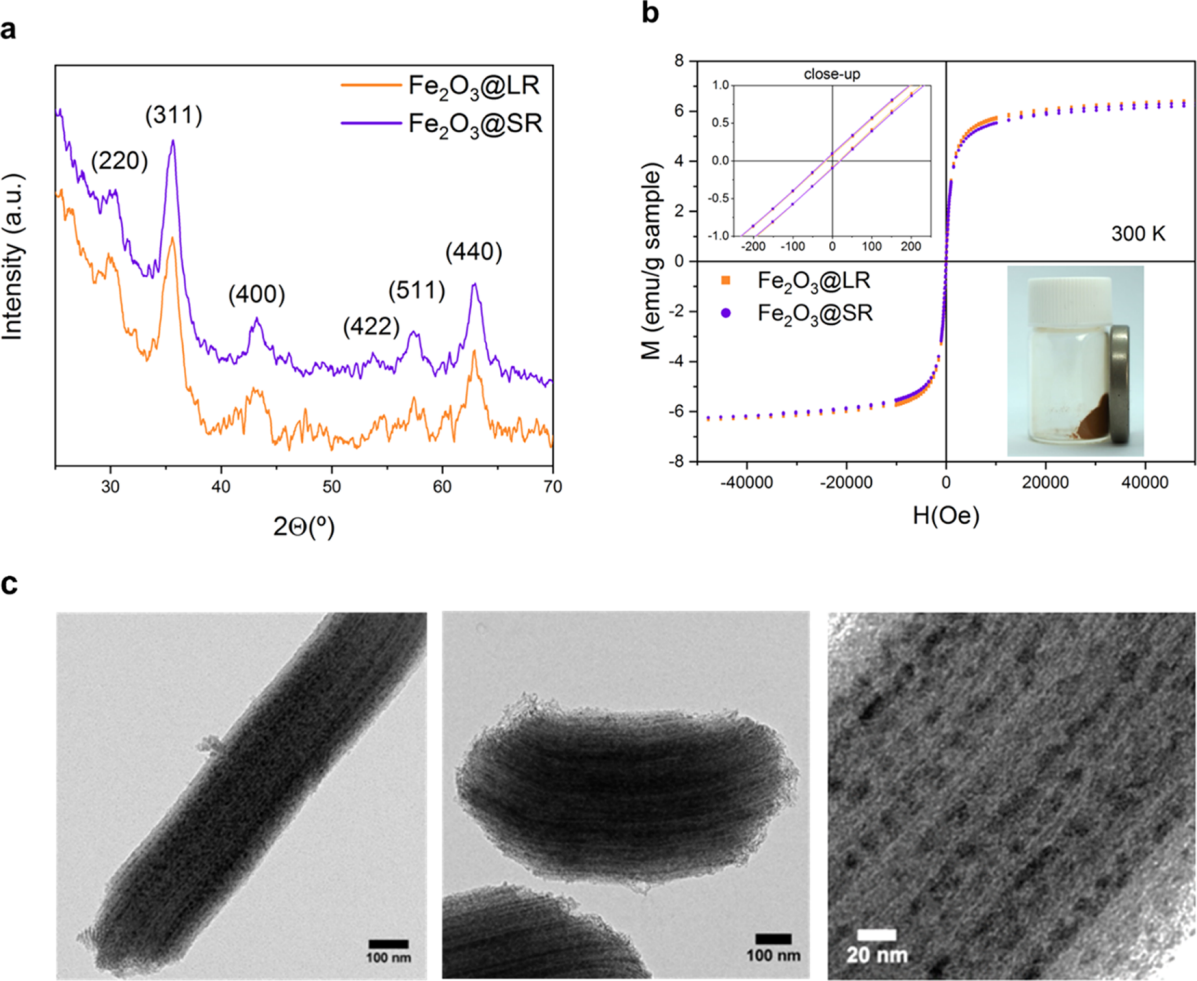
(a) XRD diffractograms of Fe_2_O_3_@LR and Fe_2_O_3_@SR. The peaks are indexed in γ-Fe_2_O_3_ unit cell. (b) Magnetic hysteresis loops of Fe_2_O_3_@LR and Fe_2_O_3_@SR at 300 K, where the magnetic moment is given per gram of sample. Inset: Image of magnetic silica rods attracted by a magnet. (c) TEM images of MSRs loaded with iron oxide nanoparticles. The leftmost image shows LR, and the central one SR. The rightmost higher-magnification image corresponds to LR, with iron oxide nanoparticles visible within the silica pores.

For the optimized annealing treatment, only maghemite peaks were observed by XRD in both materials (Figure 3a). The presence of a single phase was confirmed by Rietveld refinement analysis (Figure S7). Electron microscopy images show a large number of nanoparticles aligned inside the channels of both LR and SR (Figure 3c). The particles can be seen as slightly larger than the pores, also observed in an earlier study.^9^ To confirm that the particles locally expand the channels while growing in confinement and are not adsorbed on the outer surface of rods, the TEM stage was tilted over a wide range of angles around the long axis of the rod (Figure S8). As expected, under certain angles the channels were more visible and the particles appeared clearly aligned along with them. No particles were observed on the outer surface of the silica rods and only a very small number of empty rods were identified. The nanoparticles (NPs) induced local deformations of the pores of both types of rods as indicated by the increased pore width seen around the particles and the tortuosity of the channels along the rod axis.

The magnetization vs magnetic field at room temperature of the MSRs (Figure 3b) shows a typical superparamagnetic behavior, characterized by the absence of hysteresis in fields larger than the residual fields (in the order of 50 Oe)^62^ resulting from a trapped flux in the SQUID coils (see the inset in Figure 3b). Such behavior is characteristic for small magnetic nanoparticles (typically below 14 nm in the case of maghemite), which contain a single magnetic domain.^63^ ZFC-FC measurements (Figure S9) showed similar blocking temperatures in both Fe_2_O_3_@LR and Fe_2_O_3_@SR systems (i.e., *T*_B_ ca. 40 K) and no separation between the ZFC and FC above these temperatures, indicating a similar magnetic character of both composites and no significant magnetic interactions.

Electron diffraction studies of maghemite-loaded rods indicated that the growth of particles in confinement inside the channels induces a preferential direction. The diffraction pattern (Figure S10) indicates that the crystals have different orientations in the direction perpendicular to the rod axis but share a common preferential [110] orientation along the rod axis. In particular, all of the elongated diffraction spots of this pattern can be indexed by considering the superposition of patterns from maghemite crystals along the [1̲11], [001], [11̲0], [11̲2̲], and [3̲32] zone axes.

The periodicity and loading of the channels were studied using SAXS (Figure S11). Before loading with iron oxide NPs, three peaks were observed in the SAXS pattern and indexed as (100), (110), and (220) reflections associated with *p6mm* two-dimensional space group, indicating a hexagonal mesoscopic periodical organization characteristic of SBA-15.^7^ After loading with iron oxide NPs, the intensity of the peaks decreased as expected, and only the (100) reflection was observed with a significantly decreased intensity. This suggests that loading the pores caused a decrease in the periodicity of electron density contrast, confirming the filling of a substantial fraction of the pores.

The size and shape of NPs filling the pores were difficult to assess from the TEM images of the rods but they appear as having a slightly elongated, ellipsoidal shape. To visualize individual nanoparticles, silica was etched with a concentrated solution of sodium hydroxide. Although the particles formed aggregates, the elongated shape (6.9 nm × 4.9 nm) was confirmed in TEM measurements (Figure S12). The maghemite phase was identified using electron diffraction.

**Figure 4:**
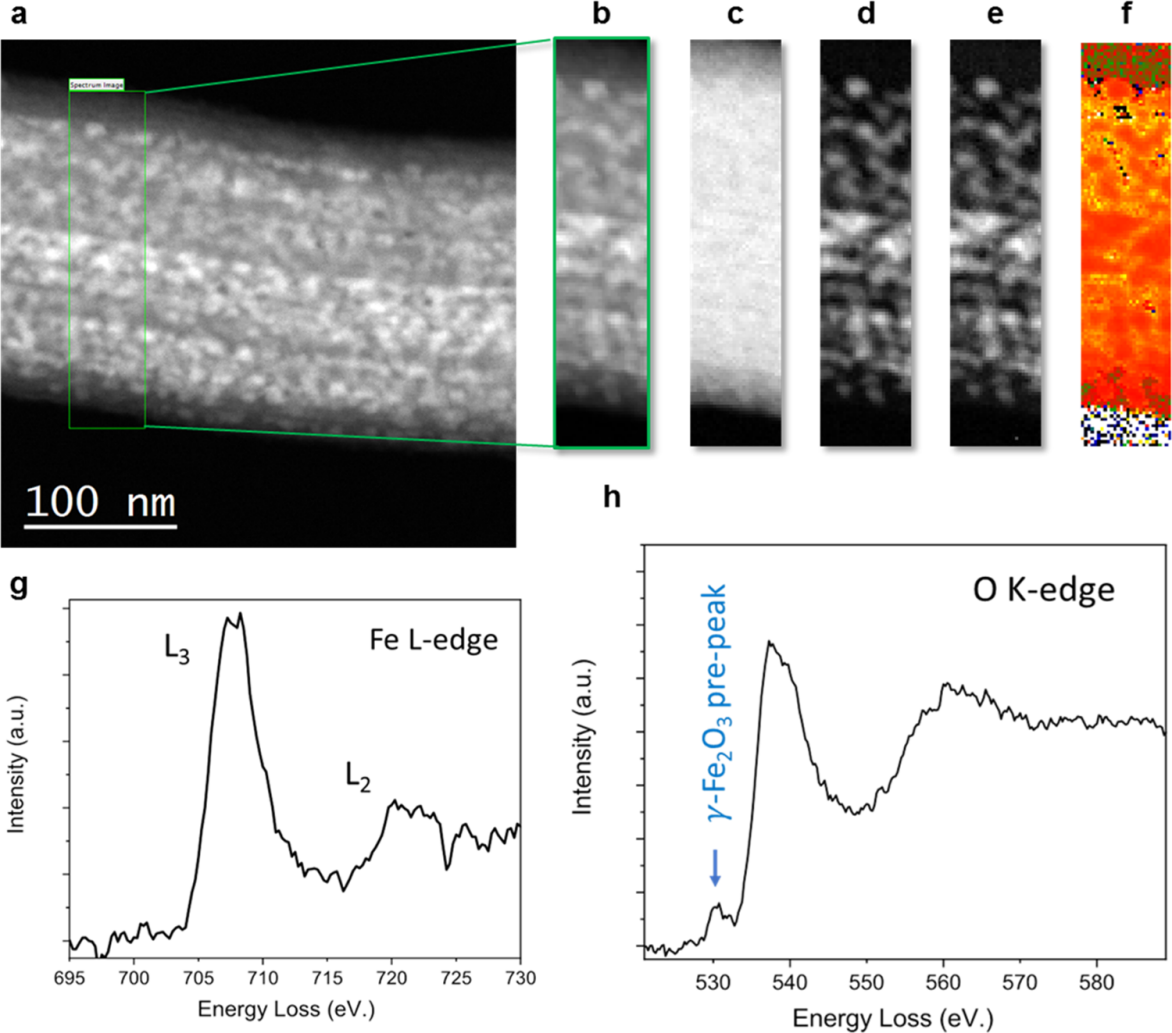
EELS data of Fe_2_O_3_@LR. (a) Zeta contrast image of a single rod. The inset shows the region where a spectrum image 28 × 127 pixels was acquired, with an exposure time of 0.1 s per pixel and an energy dispersion of 0.25 eV per channel. (b) Simultaneous annular dark-field (ADF) image of the area highlighted in the zeta contrast image. (c, d) O K-edge and Fe L-edge maps. Integration windows of width 30 eV were used after background subtraction using a power-law fit. (e) O K-edge pre-peak map obtained by integrating over a 5 eV window centered at 530 eV, after background subtraction. This pre-edge feature stems from the hybridization of O-2p and Fe-3d states of γ-iron oxide particles, and it is absent in the O K-edge signal obtained from the silica. (f) Fe L_23_ ratio map obtained using the second derivative method. The L_23_ ratio (∼5.5) is higher than that expected for Fe_3_O_4_ calculated using the same method (∼5.1).^64^ It is known that the Fe L_23_ ratio in γ-Fe_2_O_3_ is higher than that in Fe_3_O_4_.^65^ (g, h) Fe L-edge and O K-edge spectra, respectively. The O pre-peak is indicated by an arrow in the latter.

The presence of γ-Fe_2_O_3_ was also confirmed by STEM in combination with EELS (see Figure 4). The Fe L-edge map from the spectrum image coincides with the map obtained using the pre-peak feature of the O K-edge spectrum, which is characteristic for iron oxide, confirming the presence of iron oxide NPs inside the pores and excluding the presence of metallic iron. Moreover, the intensity ratio of iron L_3_/L_2_ white lines in Fe L_2,3_ spectra of approximately 5.5 indicates the presence of γ-Fe_2_O_3_ rather than Fe_3_O_4_, which presents a lower L_3_/L_2_ ratio.^64,65^

The iron oxide content was assessed using ICP-OES. The results are shown in Table 2. The iron oxide content calculated from iron content analyzed experimentally by ICP-OES agrees with the theoretical values calculated from the precursor amounts used for impregnation (i.e., ∼14 wt % Fe_2_O_3_). From these values, we expressed the saturation magnetization (*M*_*s*_) per mass of Fe_2_O_3_.

**Table 2:** Iron Oxide Content in Fe_2_O_3_@LR and Fe_2_O_3_@SR Obtained from ICP-OES Compared with the Values Calculated from the Amounts of Precursors Used for Impregnation^a^

sample ID	Fe_2_O_3_ wt % (theoretical)	Fe_2_O_3_ wt % (ICP-OES)	*M*_S_ at 300 K (emu/g Fe_2_O_3_)
Fe_2_O_3_@LR	14.3	15.0 ± 1.3	43 ± 4
Fe_2_O_3_@SR	14.3	15.0 ± 0.3	42 ± 4

a From the ICP-OES analysis, the saturation magnetization is given per gram of Fe_2_O_3_.

**Figure 5:**
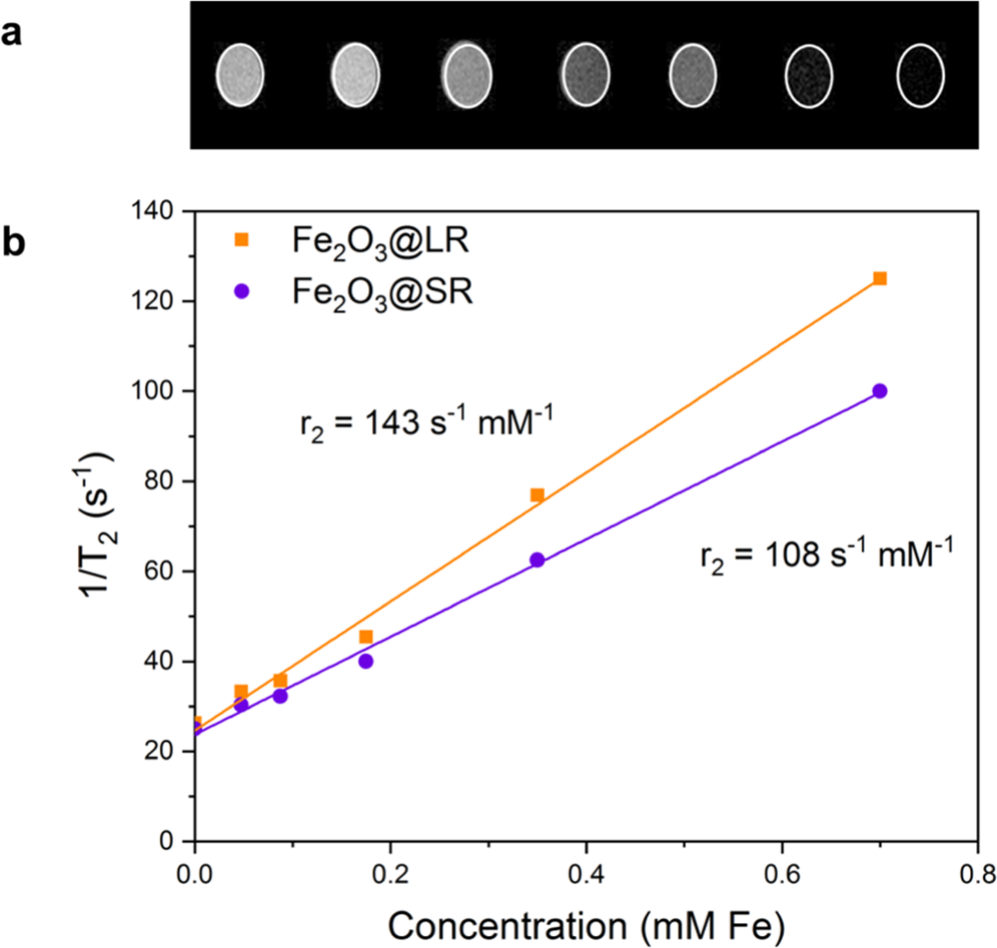
(a) T_2_ MRI signals of agarose phantoms with increasing concentrations of magnetic LR corresponding to 0, 0.175, 0.35, 0.525, 0.7, 1.75, and 3.5 mM Fe. (b) Evaluation of *r*_2_ relaxivities of Fe_2_O_3_@LR and Fe_2_O_3_@SR (*n* = 1).

Next, the performance of the magnetic composites as T_2_ MRI contrast agents was studied. Agarose phantoms containing Fe_2_O_3_@LR or Fe_2_O_3_@SR were prepared at a series of concentrations and their MRI T_2_ signal was recorded (Table S4). A decrease of the signal was observed as the concentrations of rods increased, for both long and short rods, and the inverse T_2_ relaxation time presented a linear dependence on iron concentration in the composite (Figure 5). The signal decay of long rods was higher than that of short rods, which is illustrated by their slightly higher transverse relaxivity value, *r*_2_, at 7 T (143 and 108 s^–1^ mM^–1^, respectively). The Fe_2_O_3_@LR are slightly more efficient T_2_ contrast agents because they accommodate a larger mass of Fe_2_O_3_ meaning a larger magnetic moment (emu) per particle. This causes larger magnetic field inhomogeneities in the studied vortex and a faster decrease of the time that the nuclear spins move coherently in the *x*–*y* plane.^66^

We also investigated the loading of the pores of silica rods with cerium oxide (ceria). This compound offers great potential as a therapeutic agent serving as a scavenger of reactive oxygen species (ROS) due to its redox properties.^50,67,68^ We investigated the possibility of introducing both ceria and iron oxide nanoparticles inside the rods, forming a bimodal load for potential theranostic applications. To introduce ceria inside the pores, we followed a loading approach analogous to that used for magnetic nanoparticles. For this purpose, cerium (III) nitrate hexahydrate (*T*_m_ = 65 °C) was used as a precursor. Impregnation of MSRs at 80 °C followed by a thermal treatment at 600 °C resulted in the filling of the pores with ceria, as indicated by the XRD pattern (Figure S13).

The impregnation of MSRs with the cerium precursor followed by a second impregnation with the iron precursor yielded rods appearing as filled in the TEM images (Figure S14a). It was also observed that the sample was magnetic, with the magnetization measurements indicating the presence of superparamagnetic and paramagnetic contributions (Figure S14c). However, electron diffraction (Figure S14b) and XRD (Figure S13) did not reveal the presence of any crystalline phase formed within the rods.

**Figure 6:**
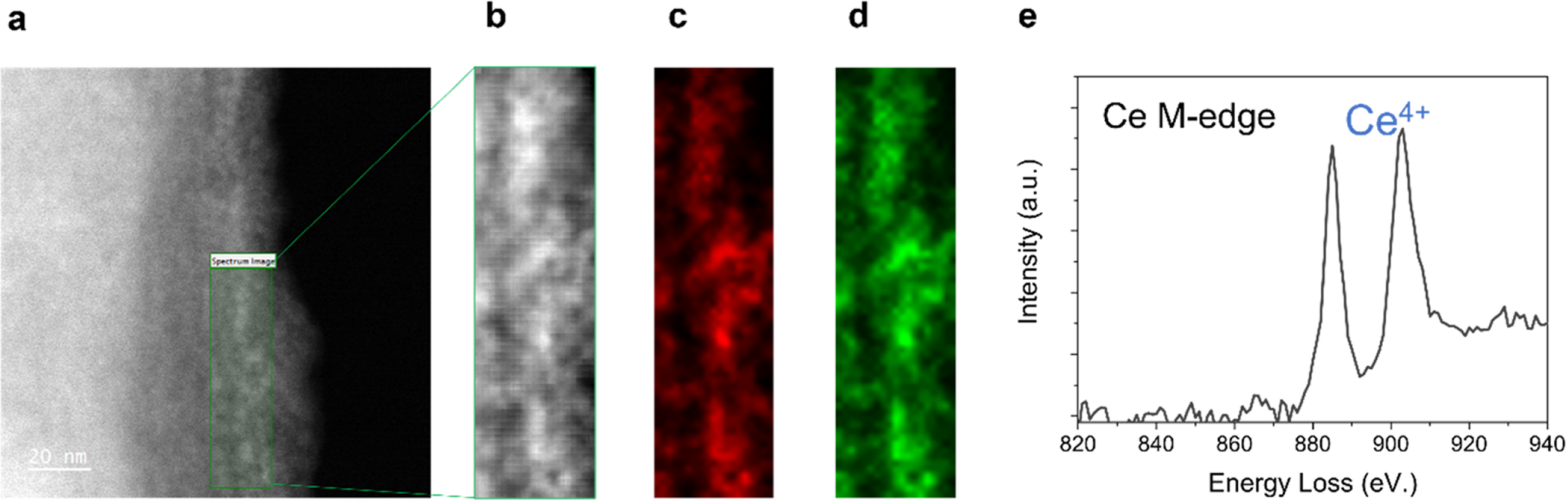
EELS spectrum at the Ce M_4,5_ edge for Fe@Ce@LR. (a) Zeta contrast image of a single rod. The inset shows the region where a spectrum image of 19 × 68 pixels was acquired, with an exposure time of 0.1 s per pixel and an energy dispersion of 1 eV per channel. (b) Simultaneous ADF image of the area highlighted in the zeta contrast image. (c, d) Fe L and Ce M-edge maps. Integration windows of width 30 eV were used after background subtraction using a power-law fit. (e) Ce M-edge signal extracted from the SI. The relative intensity of Ce M_4_ and M_5_ peaks suggests a Ce^4+^ oxidation state.^69^

To confirm the presence of both iron and cerium inside the pores and determine the oxidation state of cerium, we performed STEM analysis in combination with EELS (Figure 6). Both iron and cerium maps (Figure 6c,d) indicate that these elements are co-localized inside the pores, following the channels. Moreover, the relative intensity of cerium M_4_ to M_5_ peaks in the EEL spectrum (located at 901 and 883 eV, respectively), being M_4_ larger than M_5_, is characteristic of Ce^4+^.^69^ This indicates that ceria is in form of CeO_2_ and could be potentially used for capturing ROS.

In subsequent steps, we have explored the possibility of introducing additional functionalities to the system grafting molecular species on the surface of the silica rods. First, MSRs have been functionalized with APTES to introduce amine groups on the surface. The functionalization protocol was optimized using low APTES concentrations (6 μL/mL) and short reaction times (1 h), to avoid erosion of the rods observed at high pH. The grafting efficiency was increased by performing the reaction at an elevated temperature (70 °C), using an ethanol/water mixture as a solvent, and applying a post-grafting thermal treatment at 120 °C. After the reaction, the ζ potential measurements showed a significant shift in surface charge from negative to positive values (Figure S15a).

Moreover, TEM images (Figure S15b,c) showed that the structure of MSRs remained intact. Further evidence for change of surface character was provided from FT-IR measurements (Figure S15d). The bands corresponding to Si–O–Si symmetric stretching at 805 cm^–1^ and Si–O–Si asymmetric stretching at 1090 cm^–1^ were observed in samples both before and after functionalization, suggesting that the main structure of silica was not affected by the modification. Bands at 1610–1643 cm^–1^ observed in the functionalized silica correspond to −NH_2_ in-plane scissoring in the amine groups,^70^ while bands at 2851 and 2920 cm^–1^ indicate −CH_2_ symmetric and asymmetric stretching vibrations from the propyl branch of APTES.^71^ The positions of the bands are indicative of a dense packing of the propyl chains.^72^ An asymmetric −CH_3_ mode at 2962 cm^–1^ may suggest that some ethoxide groups have not been fully hydrolyzed and that the APTES molecules are not fully bonded with each other.^70,72^

The presence of −NH_2_ groups on the MSR surface was additionally investigated by X-ray photoelectron spectroscopy (XPS). A general scan was performed for both Fe_2_O_3_@LR-NH_2_ and Fe_2_O_3_@SR-NH_2_, followed by high-resolution scans of regions of interest: C 1s, N 1s, and O 1s. The elements present on the surface could be identified in a general scan (Figure S15e). The spectra show peaks at energies characteristic for nitrogen and carbon, introduced by APTES functionalization, as well as the silicon and oxygen from silica.^73,74^

The high-resolution spectra of the studied regions are shown in Figure S16. Because of the high width of peaks in high-resolution scans, it was not possible to distinguish chemical species with clarity. However, the shape of peaks of both N 1s and C 1s was in good agreement with data found in the literature regarding APTES-functionalized surfaces.^75,76^ The deconvolution of N 1s spectra (Figure S17) suggested surface species including C–NH_2_ and C–NH_3_^+^.^76^ The amine group (C–NH_2_) was dominant in both samples.

The introduction of amine groups to the surface of MSRs allowed us to chemically attach other molecules to obtain fluorescent materials for optical imaging. Fluorescamine, or fluram, is a nonfluorescent compound that reacts quickly with primary amines (R-NH_2_), forming a fluorescent derivative that emits green light upon irradiation with ultraviolet (UV) light (Figure S18a).^77^ Fluorescamine was conjugated to the amine groups on the surface of the APTES-functionalized MSRs. The attachment of fluram to LR and SR (yielding samples labeled as LR-FL and SR-FL, respectively) could be confirmed by fluorescence spectroscopy, considering that free fluram does not show fluorescence.

After the reaction, the color of the MSR dispersion changed from white to intense yellow (Figure S18b). Under irradiation with UV light, green fluorescence was observed (Figure S18c). The fluorescence emission spectrum was then acquired under irradiation at 390 nm, and an intense emission peak was observed at 525 nm (Figure S18d).

**Figure 7:**
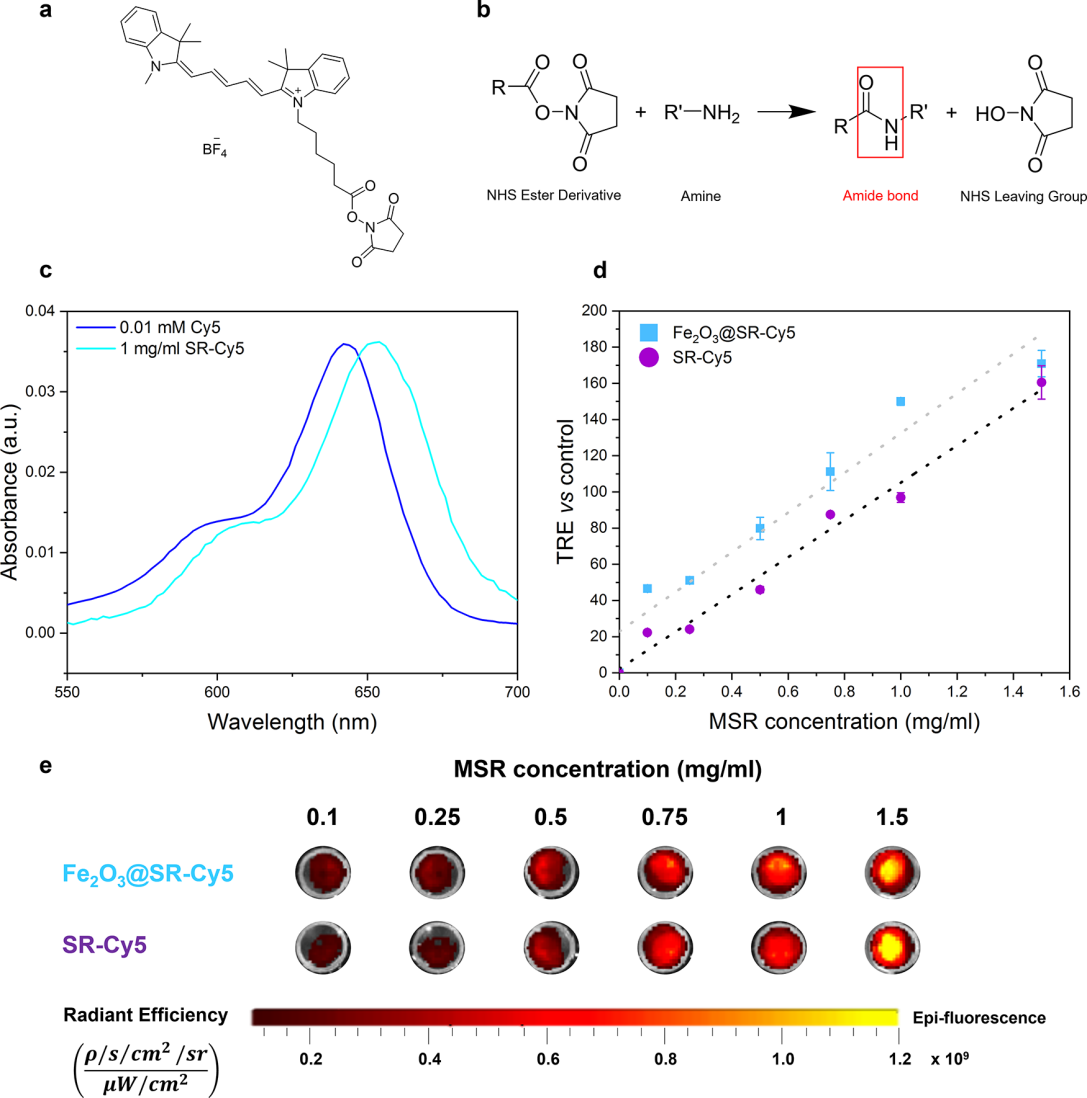
(a) Structure of Cy5-NHS ester. (b) Reaction scheme of an NHS ester derivative with a primary amine forming an amide bond. (c) Absorbance of SR-Cy5 compared to Cy5 solution. Characteristic double peak of Cy5 is observed after functionalization of silica. (d) TRE vs control of SR-Cy5 and Fe_2_O_3_@SR-Cy5 plotted as a function of sample concentration showing a linear dependence (*n* = 1). (e) Fluorescence images of SR-Cy5 and Fe_2_O_3_@SR-Cy5 dispersions for the same MSR concentrations as in (d).

MSRs were then functionalized with Cyanine5 (also described as Cy5, where Cy stands for cyanine, and the number 5 refers to the number of carbon atoms between the indolenine groups).^78^ This fluorophore emitting in the red region is often used in biomedical research for cell and tissue labeling, as in this spectral range biological objects show low absorption and many detectors exhibit maximum sensitivity.^79^ To attach the Cy5 dye to the MSR surface, N-hydroxysuccinimide (NHS) ester derivative of Cy5 (Figure 7a) was used and reacted with amine groups of APTES-functionalized silica surface, forming an amide bond (Figure 7b).^80^ After the reaction of MSRs-NH_2_ with Cy5, a blue product was observed. UV–vis absorption spectra (Figure 7c) confirmed the presence of Cy5 comparing the absorbance of free Cy5 with Cy5-functionalized SR (denominated SR-Cy5). A characteristic Cy5 band was observed at 652 nm, slightly shifted (∼10 nm) toward higher wavelengths compared to the absorbance curve of free Cy5. A similar effect has been reported in the literature for Cy5 and other fluorophores bound to surfaces^81,82^ and can be explained by the change in polarity and polarizability of the fluorophore environment compared to the free fluorophore.^83^ The fluorescence emission of SR-Cy5 and Cy5-functionalized Fe_2_O_3_@SR (Fe_2_O_3_@SR-Cy5) was studied by FMI. Optical imaging phantoms were made by preparing a dispersion of rods in an aqueous solution of d-mannitol (55 mg/mL) that was then diluted in saline to obtain a range of concentrations (0–1.5 mg/mL). Mannitol solution was used instead of water to increase the stability of the dispersion of rods in this medium. Emission was measured with the excitation filter centered at 640 nm. The particles exhibited high fluorescence, linearly increasing with the concentration (Figure 7d,e). The TRE was very similar for both SR-Cy5 and Fe_2_O_3_@SR-Cy5. Therefore, we have shown that the MSRs offer the potential of combining two imaging functional moieties without a decrease in their performance as fluorescence imaging agents.

**Figure 8:**
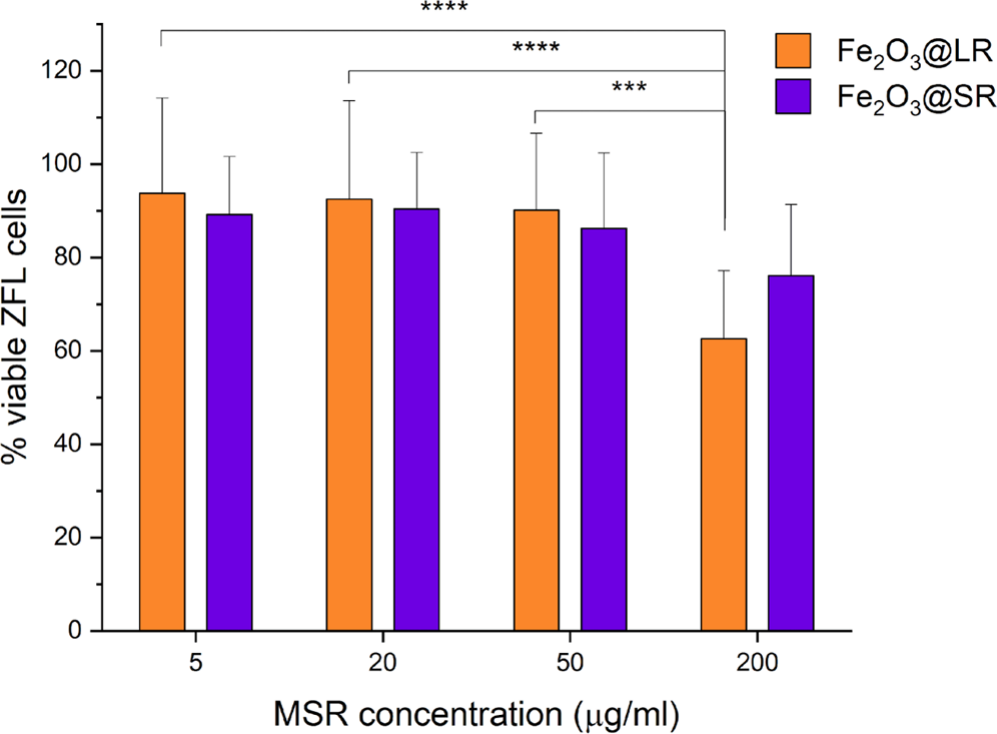
Zebrafish liver cell (ZFL) viability after 6 h of exposure to the different types of MSRs at four different concentrations; *n* = 12. ***P ≤ 0.001, ****P ≤ 0.0001.

To assess the safety of MSRs in the context of their potential use in biomedical applications, toxicity tests were performed in vitro in zebrafish liver cell line (ZFL). Neither Fe_2_O_3_@LR nor Fe_2_O_3_@SR caused cytotoxicity in ZFL cells for concentrations up to 50 μg/mL (Figure 8). At 200 μg/mL MSRs, the number of viable cells decreased significantly only for Fe_2_O_3_@LR. Due to the scarcity of studies concerning silica particles in ZFL cells, the results of toxicity studies are difficult to compare with the literature. However, these results suggest low toxicity of MSRs, especially short rods, at concentrations higher than those that have been used for carbon nanotubes.^84^

## Conclusions

We have reported on two protocols for synthesizing uniformly sized MSR with distinct aspect ratios: long rods (LR, AR = 4.7) and short rods (SR, AR = 2.2). The MSRs display an array of hexagonally ordered cylindrical pores running along the rod axis with a high surface area (up to 827 m^2^ g^–1^ for LR and 672 m^2^ g^–1^ for SR) and pore volume (up to 1 cm^3^ g^–1^ for LR and 0.8 cm^3^ g^–1^ for SR) with a pore diameter of approximately 5 nm. These channels enable the modification of the rods with chemical species. We loaded the silica pores with iron oxide nanoparticles using a wet impregnation method followed by thermal treatment. A single-step treatment in a reducing atmosphere (Ar/5% H_2_, 100 cm^3^/min) results in the formation of small particles within the silica pores, which consist of γ-iron oxide, and we confirmed that virtually no magnetic particles are formed outside the rods or on its surface. We have observed that both long and short rods filled with iron oxide nanoparticles exhibit superparamagnetic character at room temperature with a significant saturation magnetization (∼42 emu/g Fe_2_O_3_) which highlights the potential of this material to be used in biomedical applications, such as MRI imaging. The performance of the materials as T_2_-weighed MRI contrast agents has been confirmed to be slightly superior in the long rods (*r*_2_ = 143 mM^–1^ s^–1^) than short ones (*r*_2_ = 108 mM^–1^ s^–1^). The potential of the material for combining multiple functional moieties has also been confirmed by functionalizing the surface with fluorophores of distinct emission wavelengths: fluorescamine, producing a material emitting in green (525 nm), and Cyanine5, endowing the rods with red light emission (∼730 nm). Cerium oxide was additionally introduced inside the pores. It was observed that a system incorporating ceria and iron oxide in the pores presented a magnetic response but was not crystalline, indicating the presence of amorphous iron oxide co-localized with Ce^4+^ species. These multimodal magnetic materials can find applications in future theranostic developments for which the functional versatility demonstrated in this work and the therapeutic performance associated with their anisotropic shape could be advantageous over spherical silica carriers.

## Supporting Information Available

Synthesis scheme, TEM images, thermogravimetric analysis, porosity data, X-ray diffractograms, Rietveld refinements, ZFC-FC curves, electron diffraction, SAXS, MRI T_2_ relaxation times, ζ potential values, FT-IR spectra, XPS spectra, and fluorescence spectra (PDF)
