# Supplementary material for: Magnetic Mesoporous Silica Nanorods Loaded with Ceria and Functionalized with Fluorophores for Multimodal Imaging
Source: ACS Appl Nano Mater. 2022 Feb 10;5(2):2113–25. doi: 10.1021/acsanm.1c03837 (PMC8886853; doi:10.1021/acsanm.1c03837)

Supporting Information

Magnetic Mesoporous Silica Nanorods Loaded with Ceria and Functionalized with Fluorophores for Multimodal Imaging

Jan Grzelak,[a] Jaume Gázquez,[a] Alba Grayston,[b] Mariana Teles,[c] Fernando Herranz,[d] Nerea Roher,[c] Anna Rosell,[b] Anna Roig[a*], Martí Gich[a*]

a. Institut de Ciència de Materials de Barcelona (ICMAB-CSIC). Campus UAB, 08193 Bellaterra, Catalonia, Spain.

b. Neurovascular Research Laboratory Vall d'Hebron, Research Institute (VHIR), 08035, Barcelona, Catalonia, Spain.

c. Institute of Biotechnology and Biomedicine (IBB), Universitat Autònoma de Barcelona, 08193 Barcelona, Spain.

d. Instituto de Química Médica (IQM), Consejo Superior de Investigaciones Científicas (CSIC), 28006 Madrid, Spain

** corresponding authors: roig@icmab.es, marti.gich@csic.es*

Fig. S1. Upper row: Scheme depicting the synthesis of MSRs. 1) Pluronic P123 in hydrochloric acid is stirred at the reaction temperature. 2) Dropwise addition of the silica precursor. 3) In a critical step of rod fabrication, stirring is stopped after the completion of hydrolysis. 4) The condensation of silica occurs at the same temperature. 5) The bottle is transferred to an oven for the aging process at an elevated temperature. Middle row: Chemical structures of Pluronic P123 and TEOS. Hydrolysis of silica precursor. Condensation of silica. Lower row: Surfactant micelle. Hybrid cylindrical micelle. Hexagonal mesophase. Condensation of silica.

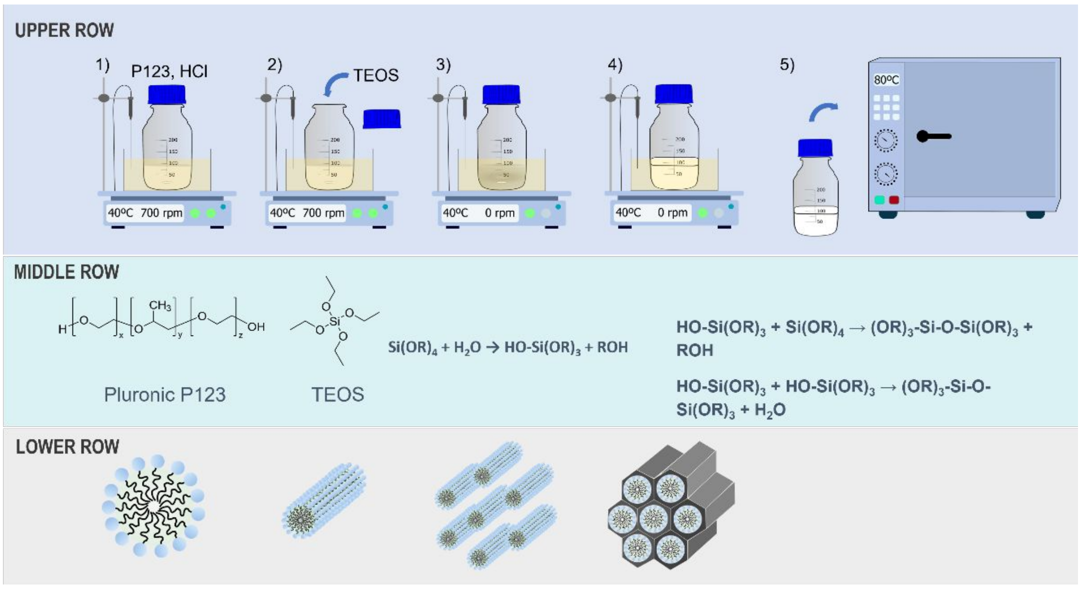

Table S1. Sets of synthesis conditions assayed: temperature (T_s), stirring rate (f) and the ratio between the volume of the reaction mixture and the volume of the bottle (V_r/V_b), in the sol-gel synthesis, and resulting silica morphologies.

Sample identification (ID)	T_s (°C)	f (rpm)	V_r/V_b	Morphology
MSN-1	40	500	0.5	branched network of rods
MSN-2	40	700	0.4	straight rods, AR ≈ 4.5
MSN-3	60	500	0.4	amorphous
MSN-4	60	700	0.5	straight rods, AR ≈ 15; tori

Fig. S2. TEM morphologies of silica particles obtained at sets of conditions described in Table S1. a) MSN-1, b) MSN-2, c) MSN-3, d) MSN-4. Scale bars: 1 μm.

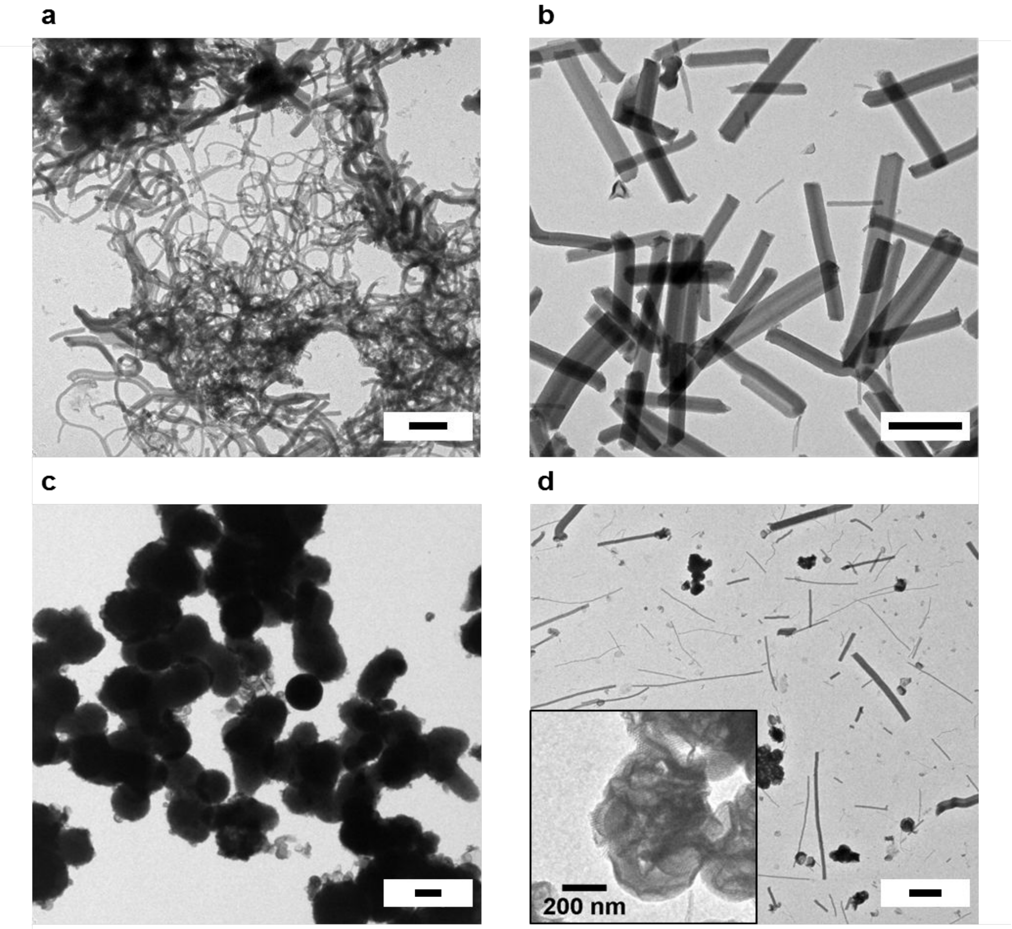

Table S2. Morphologies of silica rods obtained at various conditions. Fixed parameters: T_s = 40 °C, f = 700 rpm, *[P123]/[TEOS]* = 0.5.

Sample ID	[HCl] (M)	[P123] (mg/ml)	$\frac{V_r}{V_b}$	Length (μm)	Width (μm)	Aspect ratio
MSR1 (LR)	1.7	21	0.4	1.4 ± 0.3	0.3 ± 0.1	4.7 ± 1.3
MSR2 (SR)	2.0	30	0.3	0.9 ± 0.1	0.4 ± 0.1	2.2 ± 0.7
MSR3	1.7	30	0.4	1.2 ± 0.2	0.3 ± 0.1	4.0 ± 1.0
MSR4	2.0	21	0.3	0.8 ± 0.1	0.4 ± 0.1	2.2 ± 0.5
MSR5	1.7	30	0.3	1.0 ± 0.2	0.4 ± 0.1	2.6 ± 0.6
MSR6	2.0	21	0.4	0.9 ± 0.2	0.3 ± 0.1	3.0 ± 0.9
MSR7	1.7	21	0.3	1.4 ± 0.3	0.3 ± 0.1	5.4 ± 1.3
MSR8	2.0	30	0.4	1.0 ± 0.2	0.4 ± 0.1	2.7 ± 0.8

Fig. S3. TGA curves of LR: raw, soxhlet-cleaned and after subsequent calcination.

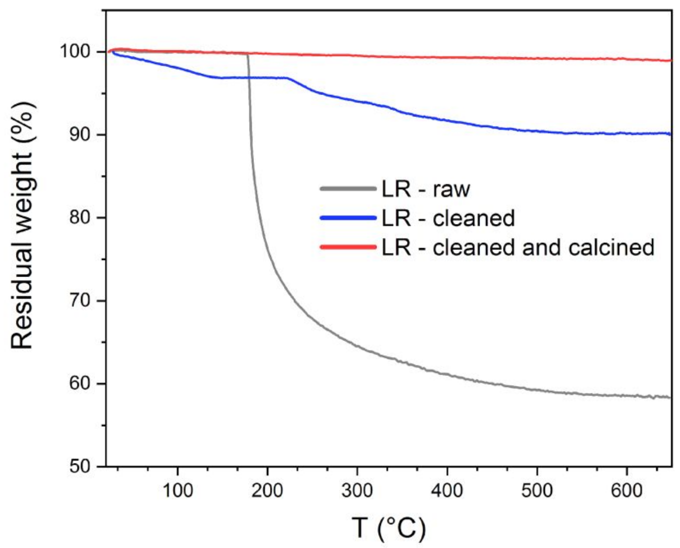

Fig. S4. (a) BET nitrogen adsorption isotherms of SR – raw, soxhlet-cleaned and calcined after cleaning. (b) BET nitrogen adsorption isotherms of LR – as-prepared, soxhlet-cleaned and calcined after cleaning. (c) Comparison of BET isotherms of LR and SR (cleaned and calcined samples used in further experiments). (d) Pore size distribution in LR and SR.

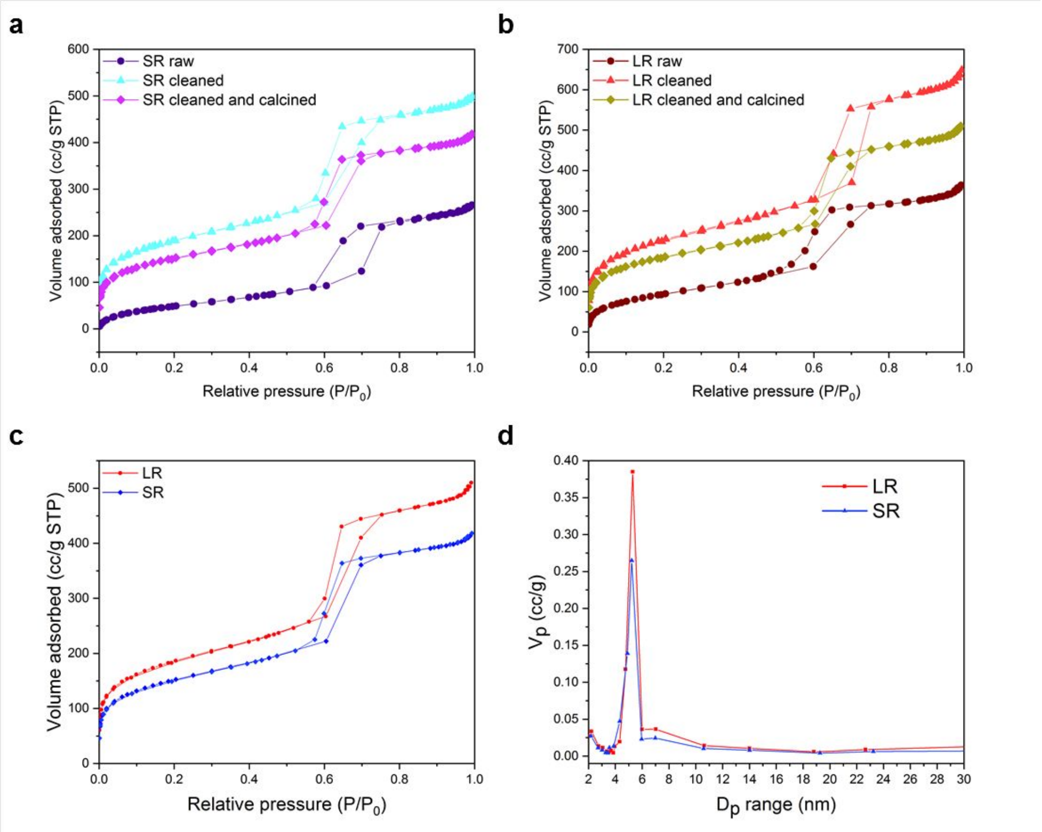

Table S3. Porosity data of MSRs obtained from nitrogen adsorption/desorption isotherms. Total BET surface area (S_{BET}), total pore volume (V_{total}), and pore diameters (D_{BJH}) obtained from the desorption branches of LR and SR. Data obtained for raw, soxhlet-cleaned (40 cycles), as well as soxhlet cleaned and calcined samples.

Sample		S_{BET} (m^2/g)	V_{total} (cc/g)	D_{BJH} (nm)
SR	Raw	188	0.41	5.5
	Cleaned	672	0.77	4.9
	Cleaned and calcined	548	0.65	5.0
LR	Raw	346	0.56	4.9
	Cleaned	827	1.00	5.5
	Cleaned and calcined	673	0.79	5.2

Fig. S5. TEM images of a calcined rod (LR), showing the uniform porosity.

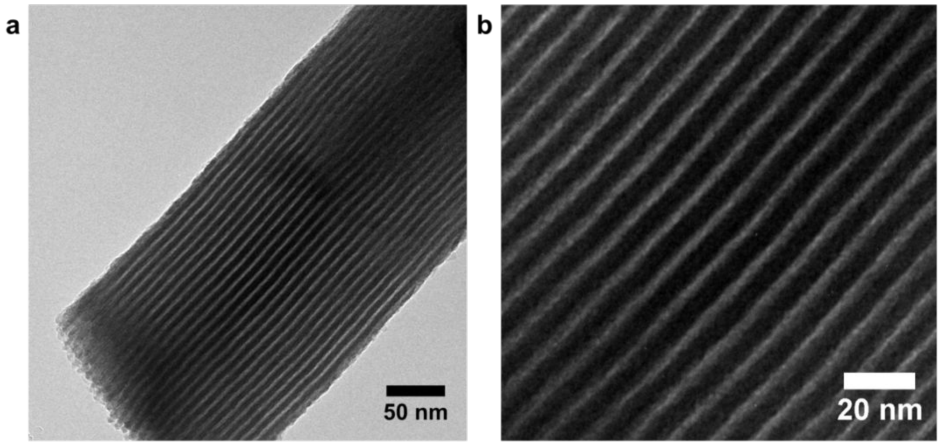

Fig. S6. X-ray diffractograms before (LR-Fe) and after (LR-Fe(Ar/H_2)) the thermal treatment in Ar/H_2. The peaks are indexed in hematite (plain text) and maghemite (underlined) unit cells. Insets: XRD theoretical patterns for maghemite (top) and hematite (bottom).

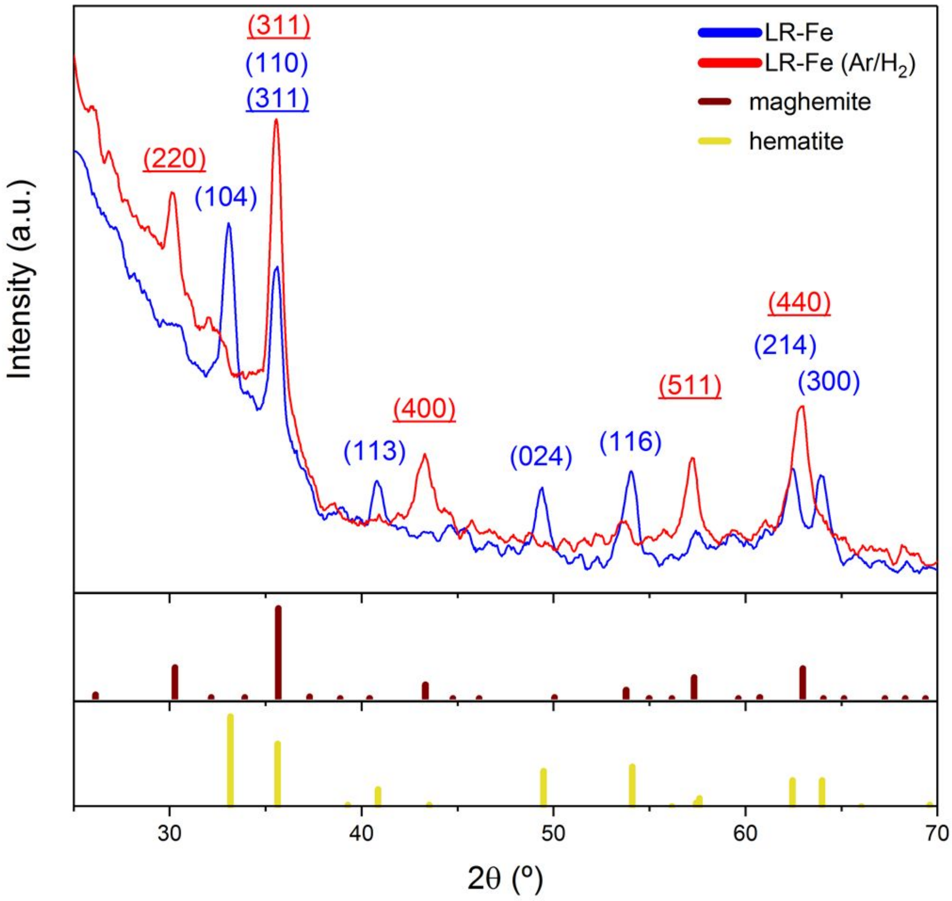

Fig. S7. Rietveld refinement results of (a) Fe_2O_3@LR and (b) Fe_2O_3@SR XRD patterns.

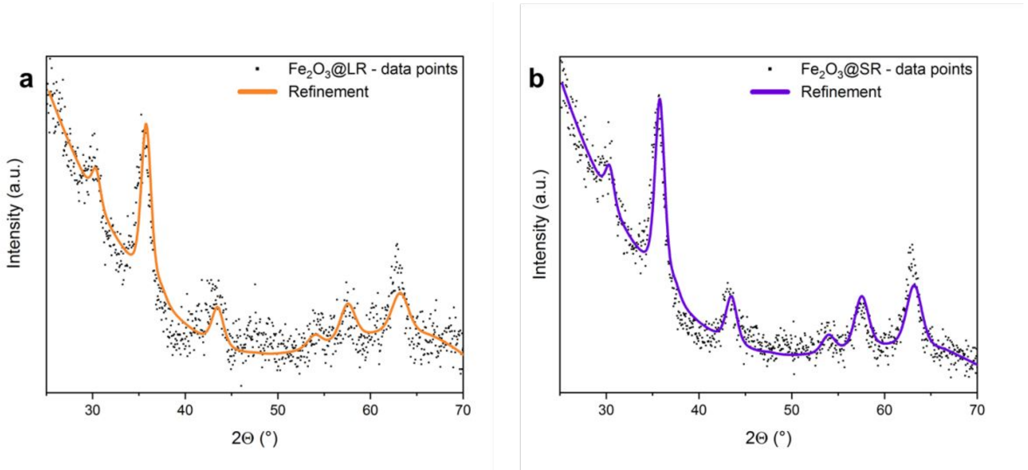

Fig. S8. (a-e) TEM images of a representative silica rod filled with γ-Fe2O3 NPs obtained after tilting the TEM stage around the rod axis. The tilt angles with respect to first image (a) are: (b) 2.8 °, (c) 5.5 °, (d) 8.9 °, (e) 14.8 °. (f) A TEM image of an empty rod for comparison.

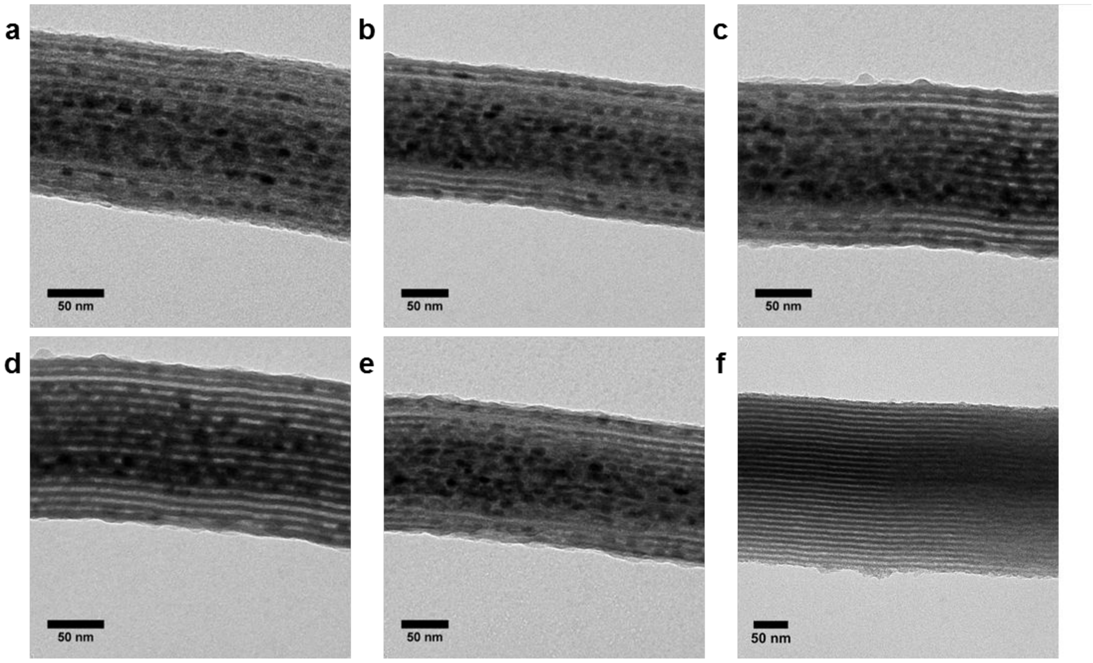

Fig. S9. ZFC-FC curves of Fe_2O_3@LR and Fe_2O_3@SR measured at 50 Oe.

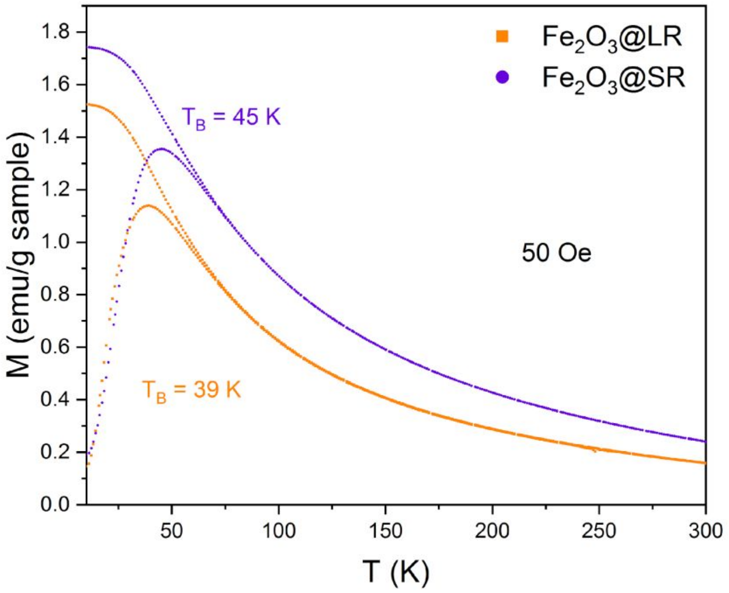

Fig. S10. Electron diffraction pattern obtained from Fe_2O_3@LR indexed considering the superposition of crystals with orientations along the [**1**11], [001], [1**1**0], [1**12**] and [**3**32] zone axes, indicated in different colors. The parentheses in the Miller indices were omitted for the clarity of the image. Inset: Corresponding TEM image of the rod.

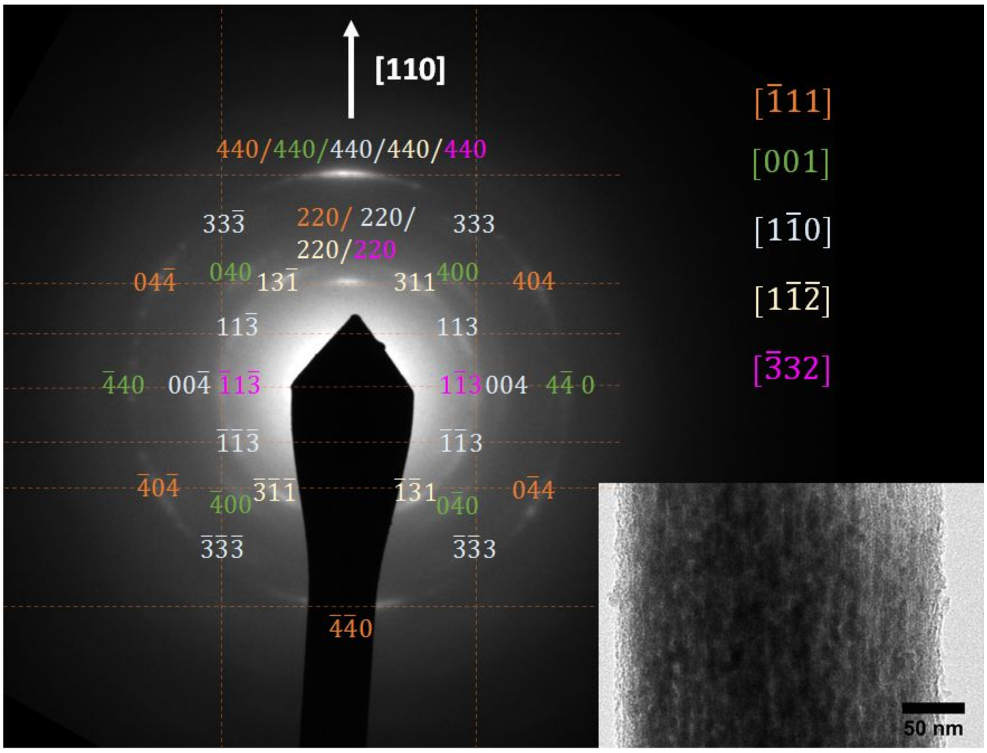

Fig. S11. SAXS patterns of LR and Fe_2O_3@LR. Scattering reflections are indexed in a hexagonal unit cell of the *p6mm* space group.

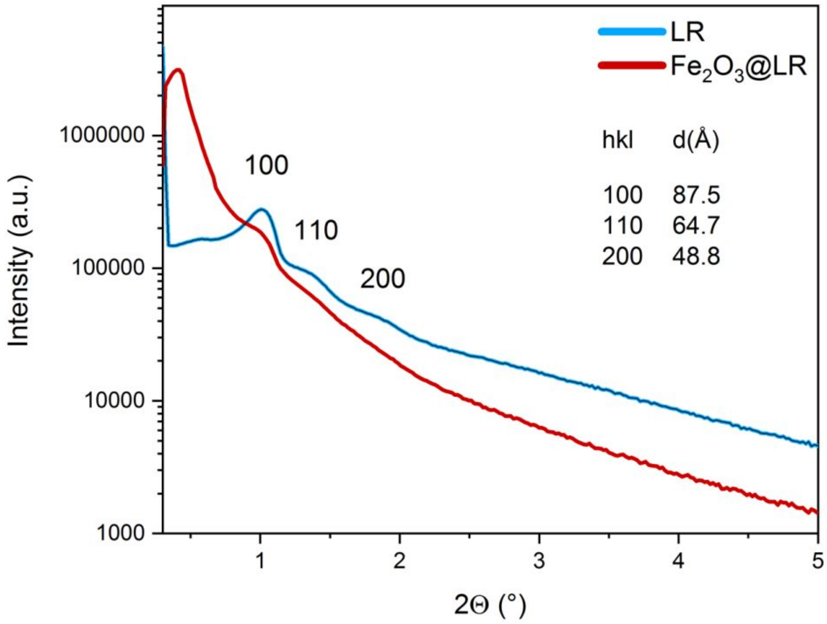

Fig. S12. (a) TEM image of iron oxide nanoparticles obtained after silica etching in Fe_2O_3@LR. (b) Corresponding electron diffraction pattern with rings indexed in maghemite unit cell. (c) A magnification of the TEM image shows an ellipsoidal shape of the nanoparticles. (d) Size distribution of the nanoparticles (n = 220) fit with a Gaussian distribution.

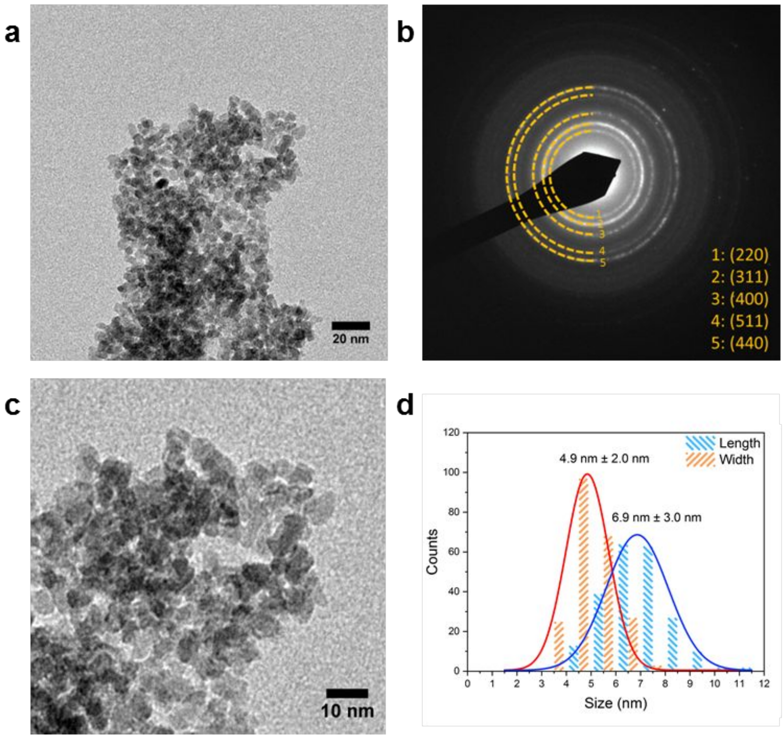

Table S4. MRI T_2 relaxation times (expressed as T_2, ms) of the MSR agarose phantoms at increasing concentrations.

Nanorods suspension		Fe_2O_3@LR	Fe_2O_3@SR
Agarose (wt. %)	Concentration (mM Fe)	T_2 (ms)	
4	0	38	40
4	0.044	30	33
4	0.088	28	31
4	0.175	22	25
4	0.350	13	16
4	0.700	8	10

Fig. S13. X-ray diffractogram of samples impregnated with cerium precursor (Ce@MSR) and with cerium precursor followed by iron precursor (Fe@Ce@MSR), indexed in the cerium (IV) oxide unit cell.

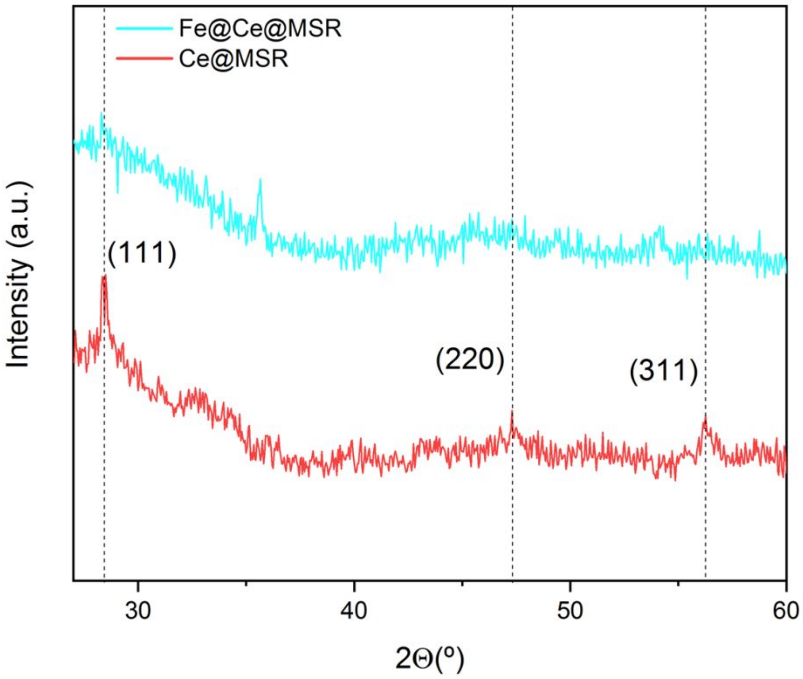

Fig. S14. (a) TEM image of Fe@Ce@MSR. (b) Corresponding electron diffraction pattern. (c) Magnetic hysteresis loop of the sample, acquired at 10K. Inset: magnification of the curve at low magnetic fields.

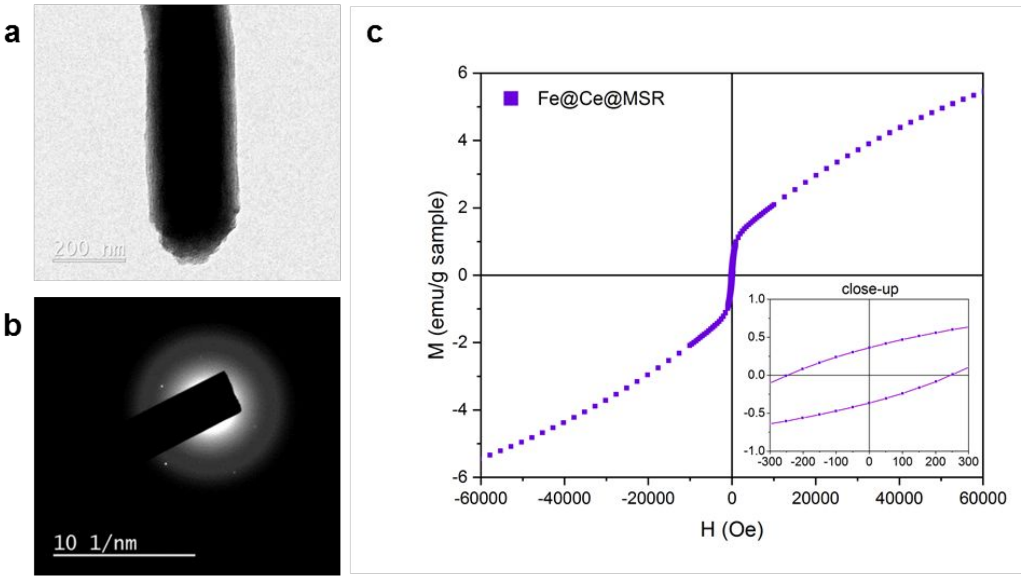

Fig. S15. (a) Zeta potential values before and after the functionalization. (b), (c) TEM images of MSRs after APTES functionalization using the final protocol. Scale bars: 50 nm. (d) FT-IR spectra of iron oxide loaded MSRs (Fe_2O_3@SR) before and after surface functionalization. (e) General XPS spectra of Fe_2O_3@SR-NH_2 and Fe_2O_3@LR-NH_2.

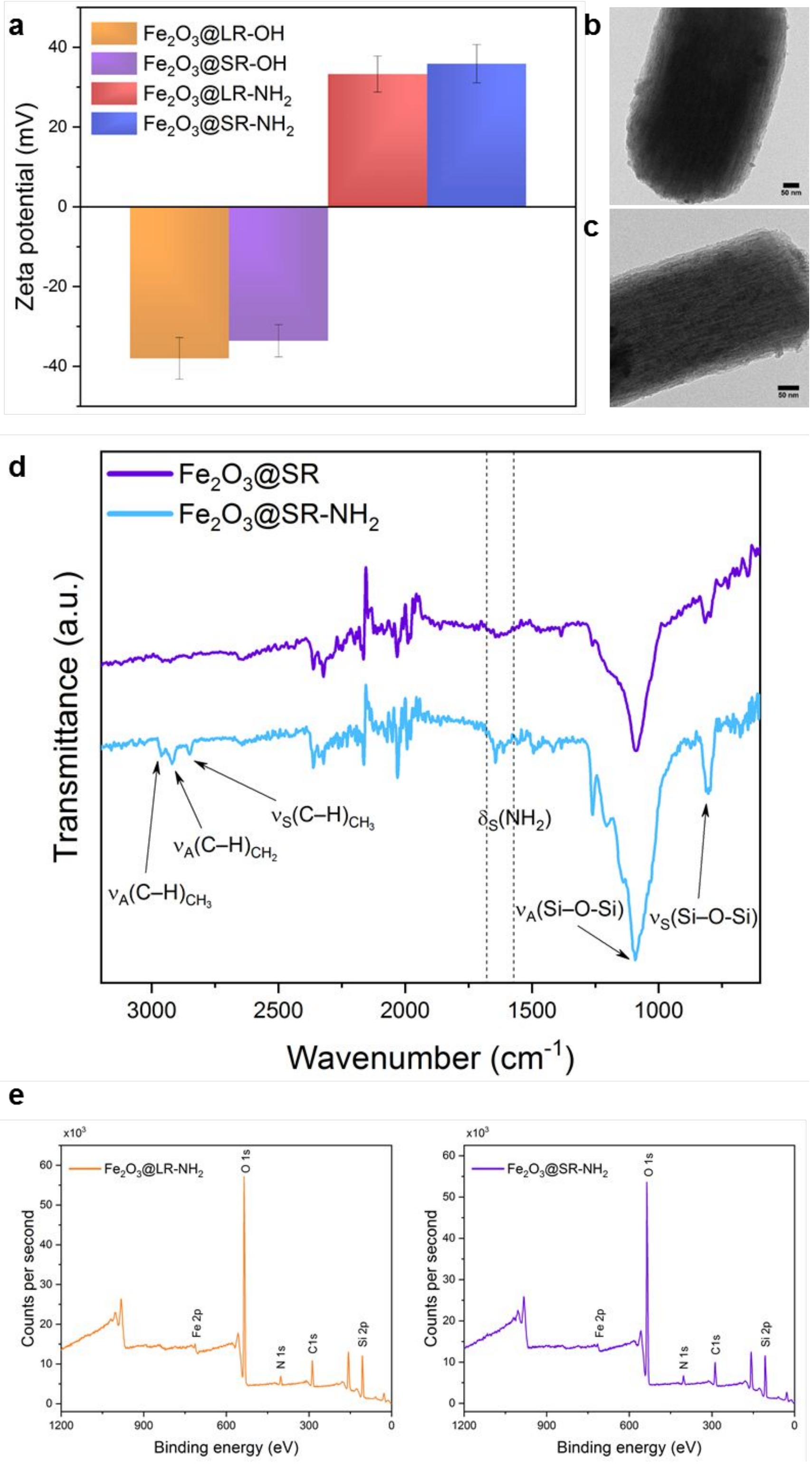

Fig. S16. High resolution XPS spectra of regions of interest of APTES-functionalized rods filled with iron oxide NPs: Fe_2O_3@LR-NH_2 and Fe_2O_3@SR-NH_2: (a) Si 2p, (b) O 1s, (c) C 1s, and (d) N 1s.

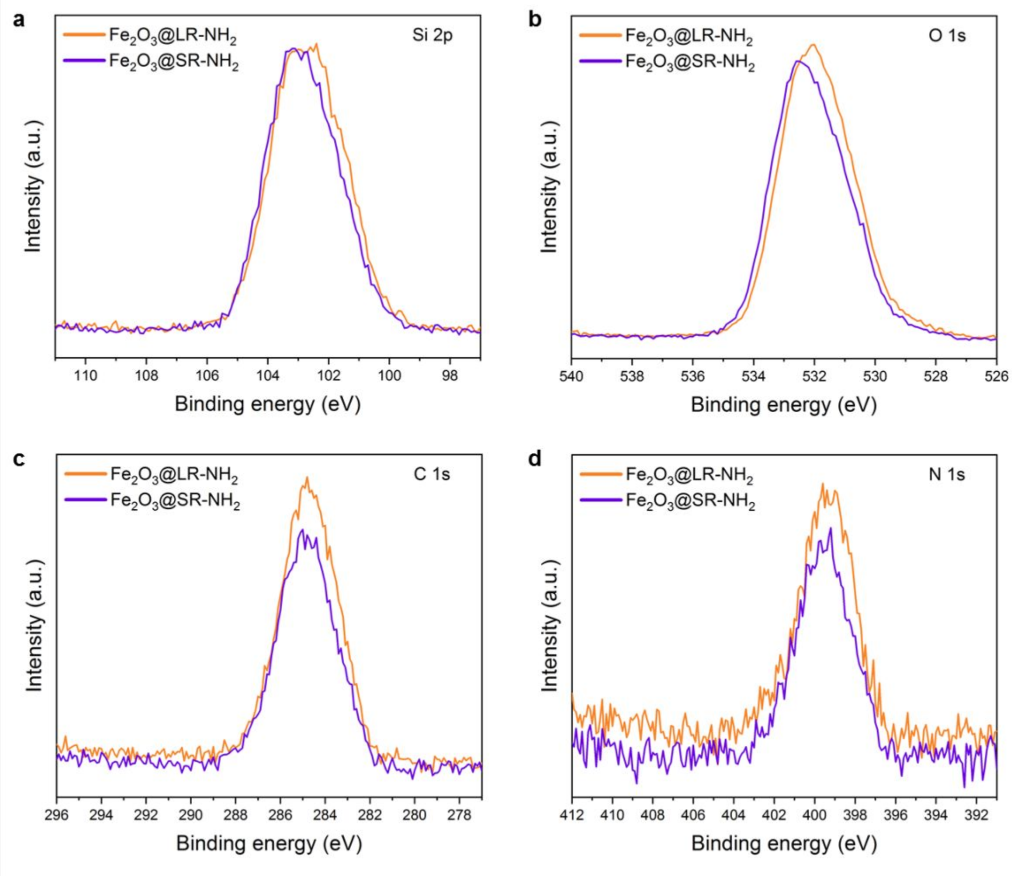

Fig. S17. Surface species classification in N 1s spectra of Fe_2O_3@LR-NH_2 and Fe_2O_3@SR-NH_2.

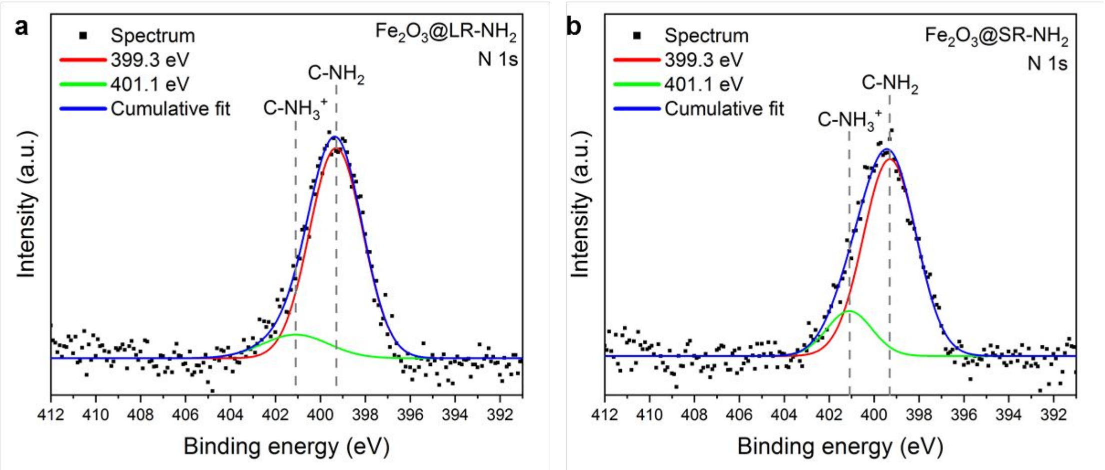

Fig. S18. (a) The structure of fluorescamine and the fluorescent compound formed as a result of its reaction with primary amines. (b) Dispersions of LR-FL and SR-FL under white light. (c) Dispersions of LR-FL and SR-FL under UV light. (d) Fluorescence emission spectra of LR-FL and SR-FL under excitation at 390 nm.

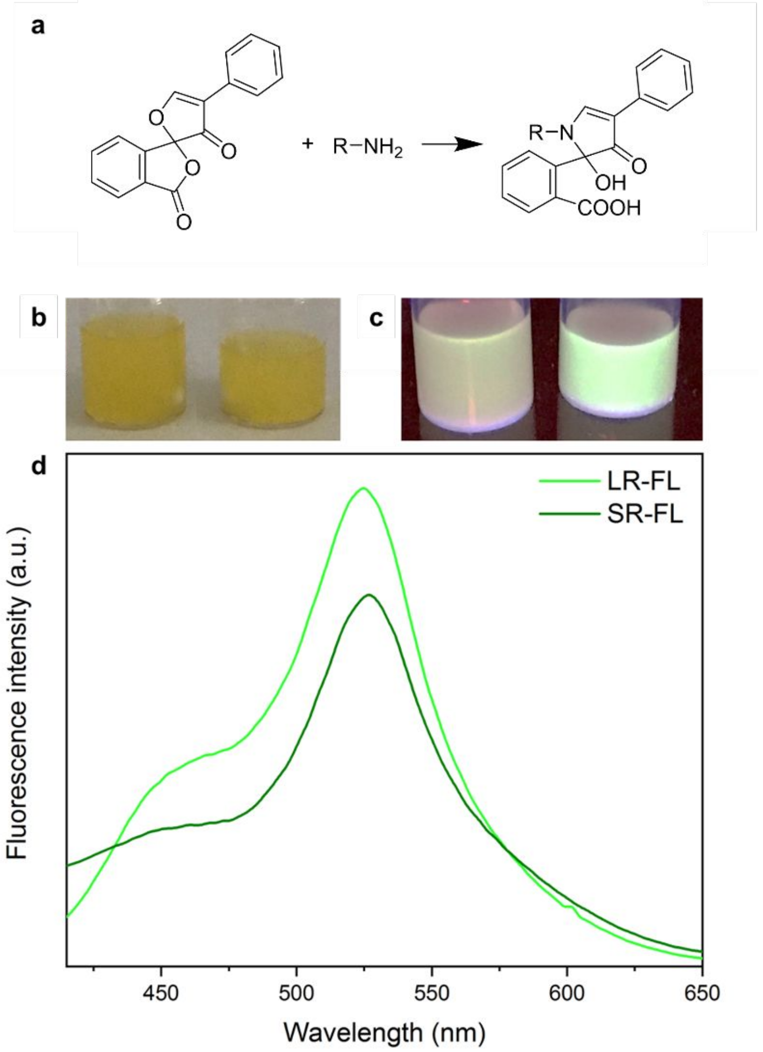

Supplement: Supplementary file 1 — an1c03837_si_001.pdf [file an1c03837_si_001.pdf]
